# A Web-Based mHealth Intervention With Telephone Support to Increase Physical Activity Among Pregnant Patients With Overweight or Obesity: Feasibility Randomized Controlled Trial

**DOI:** 10.2196/33929

**Published:** 2022-06-22

**Authors:** Tainayah Thomas, Fei Xu, Sneha Sridhar, Tali Sedgwick, Linda Nkemere, Sylvia E Badon, Charles Quesenberry, Assiamira Ferrara, Sarah Mandel, Susan D Brown, Monique Hedderson

**Affiliations:** 1 Department of Epidemiology and Population Health Stanford University School of Medicine Palo Alto, CA United States; 2 Division of Research, Kaiser Permanente Northern California Oakland, CA United States; 3 The Permanente Medical Group San Francisco, CA United States; 4 School of Medicine, University of California, Davis Sacramento, CA United States; 5 Kaiser Permanente Bernard J. Tyson School of Medicine Pasadena, CA United States

**Keywords:** mobile health, gestational weight gain, obesity, physical activity, mobile phone

## Abstract

**Background:**

Pregnant patients with overweight or obesity are at high risk for perinatal complications. Excess gestational weight gain (GWG) further exacerbates this risk. Mobile health (mHealth) lifestyle interventions that leverage technology to facilitate self-monitoring and provide just-in-time feedback may motivate behavior change to reduce excess GWG, reduce intervention costs, and increase scalability by improving access.

**Objective:**

This study aimed to test the acceptability and feasibility of a pilot mHealth lifestyle intervention for pregnant patients with overweight or obesity to promote moderate intensity physical activity (PA), encourage guideline-concordant GWG, and inform the design of a larger pragmatic cluster randomized controlled trial.

**Methods:**

We conducted a mixed methods acceptability and feasibility randomized controlled trial among pregnant patients with a prepregnancy BMI of 25 to 40 kg/m^2^. Patients with singletons at 8 to 15 weeks of gestation who were aged ≥21 years and had Wi-Fi access were recruited via email from 2 clinics within Kaiser Permanente Northern California and randomized to receive usual prenatal care or an mHealth lifestyle intervention. Participants in the intervention arm received wireless scales, access to an intervention website, activity trackers to receive automated feedback on weight gain and activity goals, and monthly calls from a lifestyle coach. Surveys and focus groups with intervention participants assessed intervention satisfaction and ways to improve the intervention. PA outcomes were self-assessed using the Pregnancy Physical Activity Questionnaire, and GWG was assessed using electronic health record data for both arms.

**Results:**

Overall, 33 patients were randomly assigned to the intervention arm, and 35 patients were randomly assigned to the usual care arm. All participants in the intervention arm weighed themselves at least once a week, compared with 20% (7/35) of the participants in the usual care arm. Participants in the intervention arm wore the activity tracker 6.4 days per week and weighed themselves 5.3 times per week, and 88% (29/33) of them rated the program “good to excellent.” Focus groups found that participants desired more nutrition-related support to help them manage GWG and would have preferred an app instead of a website. Participants in the intervention arm had a 23.46 metabolic equivalent of task hours greater change in total PA per week and a 247.2-minute greater change in moderate intensity PA per week in unadjusted models, but these effects were attenuated in adjusted models (change in total PA: 15.55 metabolic equivalent of task hours per week; change in moderate intensity PA: 199.6 minutes per week). We found no difference in total GWG (mean difference 1.14 kg) compared with usual care.

**Conclusions:**

The pilot mHealth lifestyle intervention was feasible, highly acceptable, and promoted self-monitoring. Refined interventions are needed to effectively affect PA and GWG among pregnant patients with overweight or obesity.

**Trial Registration:**

ClinicalTrials.gov NCT03936283; https://clinicaltrials.gov/ct2/show/NCT03936283

## Introduction

### Background

Pregnant patients with overweight or obesity (BMI ≥25 kg/m^2^) are at high risk for perinatal complications, including gestational diabetes, pre-eclampsia, excess fetal growth, birth injuries, and cesarean section [1,2]. Excess gestational weight gain (GWG) further exacerbates the elevated risk of perinatal complications in pregnant patients with overweight or obesity. More than half of pregnant patients with overweight or obesity exceed the recommended amount of GWG [3,4].

Pregnancy is a unique window in which patients are often motivated to make healthy changes, which presents an unparalleled opportunity to intervene in health behaviors. Intensive behavioral interventions requiring in-person counseling with multiple clinic visits may not be feasible for many patients. Technology such as mobile health (mHealth) interventions can deliver automated, standardized information that eliminates social barriers [5-7], while potentially reducing intervention costs [8-12] and improving quality [10,13-15].

Usual prenatal care includes regular weight measurements at prenatal care visits; however, during early pregnancy, these visits are infrequent, and patients may gain more weight between visits than that suggested by the Institute of Medicine (IOM) GWG guidelines. This makes it critical to assess the impact of promoting self-weighing between visits and sharing this information with the patients’ clinical care team to better evaluate and support guideline-concordant weight gain.

Research has shown that physical activity (PA) alone can reduce GWG [16-19]. For example, a meta-analysis of 12 intervention trials assessing the association between PA during pregnancy and GWG found a significantly lower average GWG in the intervention group than in the control group [19]. Current guidelines recommend pregnant patients get ≥30 minutes per day of moderate intensity PA, most days of the week [20], but this goal is rarely achieved [21]. Therefore, interventions to improve PA, especially among pregnant patients with overweight and obesity are needed. Commercially available technologies such as activity trackers (shown to increase PA in nonpregnant adults [22]) and wireless scales enable real-time self-monitoring, goal setting, and tailored feedback on goals. Tailored feedback has been successful in reinforcing motivation for behavior change, especially when delivered in relation to goal attainment [23-25]. Using technology to facilitate behavior change has the advantage of providing a resource that patients can use conveniently without disrupting their busy lives. In addition, self-monitoring of weight with wireless scales can transmit weight data directly to health coaches and clinicians, allow for objective measurement of daily weight, increase adherence compared with paper monitoring [26], and promote weight loss in adults with overweight BMI when used in conjunction with additional behavior change techniques [27]. Although wireless scales and activity trackers may facilitate self-monitoring, provide just-in-time feedback, and motivate change, few studies have both quantitatively and qualitatively evaluated the feasibility, acceptability, and efficacy of technology-based mHealth lifestyle interventions in pregnant patients with overweight or obesity.

### Objectives

This pilot acceptability and feasibility randomized controlled trial was developed to inform the design of a larger pragmatic cluster randomized controlled trial. The objective of this mixed methods study was to test the acceptability and feasibility of a pilot mHealth lifestyle intervention for pregnant patients with overweight or obesity to promote self-monitoring of weight and PA and encourage guideline-concordant GWG. As such, the primary aim was to investigate whether it was feasible to implement the intervention with the target population. The study also assessed the perceived usefulness of the intervention to determine whether it was acceptable to the study participants. Finally, the pilot acceptability and feasibility randomized controlled trial explored the preliminary efficacy findings (ie, PA and GWG) using adjusted intention-to-treat analyses.

## Methods

### Study Design

*Study of a Randomized Intervention Designed to Increase Exercise in Pregnancy* (STRIDE) is a 2-arm, parallel group pilot randomized controlled trial (ClinicalTrials.gov identifier: NCT03936283) conducted between May 2017 and May 2018. We used a mixed methods study design to assess the acceptability and feasibility of the STRIDE mHealth lifestyle intervention in a sample of pregnant patients with overweight or obesity from 2 Kaiser Permanente Northern California (KPNC) medical clinics. Participants were randomized to receive usual care (n=35) or usual care plus an mHealth lifestyle intervention (n=33). Participants completed a web-based survey at 10 and 33 weeks of gestation. To better understand the perspectives of intervention participants, participants in the intervention arm completed a program evaluation survey (n=33) and participated in 3 focus groups (n=14).

### Ethics Approval

STRIDE was approved by the KPNC Institutional Review Board (approval number 1278778).

### Eligibility

Pregnant patients who were at <12 gestational weeks and received care at KPNC medical centers were first identified in the electronic health record (EHR). Eligibility criteria were as follows: (1) aged ≥21 years; (2) prepregnancy BMI between 25 and 40 kg/m^2^ (based on weight measured in the clinical setting within 12 months before the last menstrual period, or if unavailable, the first weight measured within the first 10 weeks of pregnancy); and (3) a singleton pregnancy. Eligibility was further assessed through a tiered process beginning with approval from medical providers to contact each patient and EHR review. Medical exclusion criteria that may affect outcome assessment, evaluated by the EHR review and interview during a recruitment screening call, included multiple gestation, pregnancy loss, high-risk pregnancy (ie, drug or alcohol abuse, chronic health problems, or pregnancy complications), thyroid disease diagnosed in the last 30 days, and use of glucose-lowering medications or corticosteroids. Exclusion criteria that may interfere with full participation in the trial were assessed starting at the recruitment call and included plans to move out of the area or change health plan membership during pregnancy, no reliable access to a smartphone and Wi-Fi at home, inability to communicate in English, and unwillingness to be randomized.

### Randomization, Recruitment, and Masking

**Figure 1:**
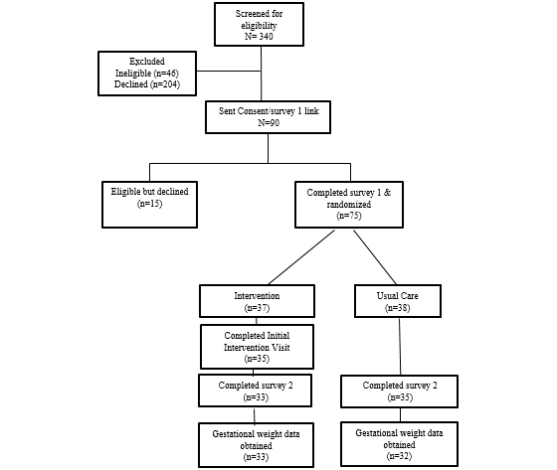
Flowchart of the Study of a Randomized Intervention Designed to Increase Exercise in Pregnancy (STRIDE).

Patients were randomly assigned to the mHealth lifestyle intervention arm or the usual care control arm upon completion of a consent form and survey 1 (Figure 1). The adaptive randomization procedure ensured that equivalent numbers of patients were assigned to each study arm and that the 2 study arms remained balanced overall and at each level of key characteristics: age (21-29.9, 30-34.9, and ≥35 years), prepregnancy BMI (25.0-29.9, 30.0-34.9, and 35.0-39.9 kg/m^2^), and race and ethnicity (Asian or Pacific Islander, Black, Hispanic, White, and multiethnic or other or unknown). Study participants were recruited via email by the study staff with a follow-up phone call 1 week later. The biostatistician, clinicians, and research assistants (LN and Socorro Dalton) who sent out study-related emails and surveys and investigators were masked to study arm assignment.

### Usual Care

Participants were randomized to the usual care arm and received standard KPNC prenatal medical care. This includes an initial prenatal visit at 7 to 10 weeks of gestation and a newsletter containing the IOM GWG guidelines and advice on healthy eating. Participants with routine pregnancies received an additional 7 prenatal visits between 16 weeks of gestation and delivery. Medical staff weighed the patients at each visit per the standard care.

### Intervention

In addition to the aforementioned usual care, patients randomized to the intervention arm received a multicomponent mHealth lifestyle intervention. The intervention targeted behavior changes for PA and weight management to help patients gain within the IOM recommended range for GWG according to their prepregnancy BMI category (7-11.5 kg for women with overweight and 5-9 kg for women with obesity [28]). Our mHealth pilot intervention was adapted from the Gestational Weight Gain and Optimal Wellness (GLOW) trial, a theory-based behavioral intervention that adapted the National Diabetes Prevention Program [29], and was delivered primarily by telehealth, for pregnant patients with overweight or obesity with the goal of reducing excess GWG [30]. The GLOW intervention consisted of 2 in-person and 11 telephone sessions on behavioral strategies to improve weight management, PA, diet, and stress management in addition to usual antenatal care. Compared with usual care only, the GLOW intervention substantially reduced the proportion of participants exceeding the IOM guidelines for weekly rate of GWG and reduced total caloric intake, proportion of calories from saturated fat, sedentary behaviors, serum leptin concentration, and markers of insulin resistance among intervention participants [30]. This pilot mHealth intervention aimed to build upon the GLOW trial and incorporate its successful components into a mobile modality.

### Conceptual Framework for the Intervention

We followed a tailored, trimester-specific approach to behavior change using constructs from social cognitive theory by Bandura [31-33] and the transtheoretical model [34], which have been the basis of adherence to healthy diet and PA in past research [30,35-37]. Key components included in the mHealth tool were as follows: (1) self-monitoring: weight self-monitoring enhances weight management [38,39] and (2) goal setting: participants were encouraged to set sequential, realistic, and short-term PA goals.

### Behaviors Targeted by the Intervention

#### PA Goals

Participants in the intervention arm were asked to set PA goals and gradually increase their activity to ultimately reach 150 minutes of moderate to vigorous activity per week, in accordance with the current American College of Obstetricians and Gynecologists recommendations [20]. Participants were provided with a Withings Activité Pop PA tracker that was worn on the wrist and tracked daily steps and minutes of moderate to vigorous intensity PA (MVPA) based on a 3-axis accelerometer optoelectronics sensor with intensity based on a metabolic coefficient ≥3. Withings activity trackers are among the most accurate for measuring steps and MVPA [40,41], with the lowest rate of false positive steps [42]. The participants were encouraged to wear their tracker daily.

#### Self-weighing

Participants were provided with a Withings Body digital scale that transmits weights via Wi-Fi or Bluetooth and has a margin of error on weight <200 g against a gold standard scale [43]. The participants were encouraged to weigh themselves daily at home.

#### Healthy Eating

Patients who desired a tool to track their diet were referred to popular free mHealth apps and websites such as MyFitnessPal and Choose MyPlate.

### Intervention Components

**Figure 2:**
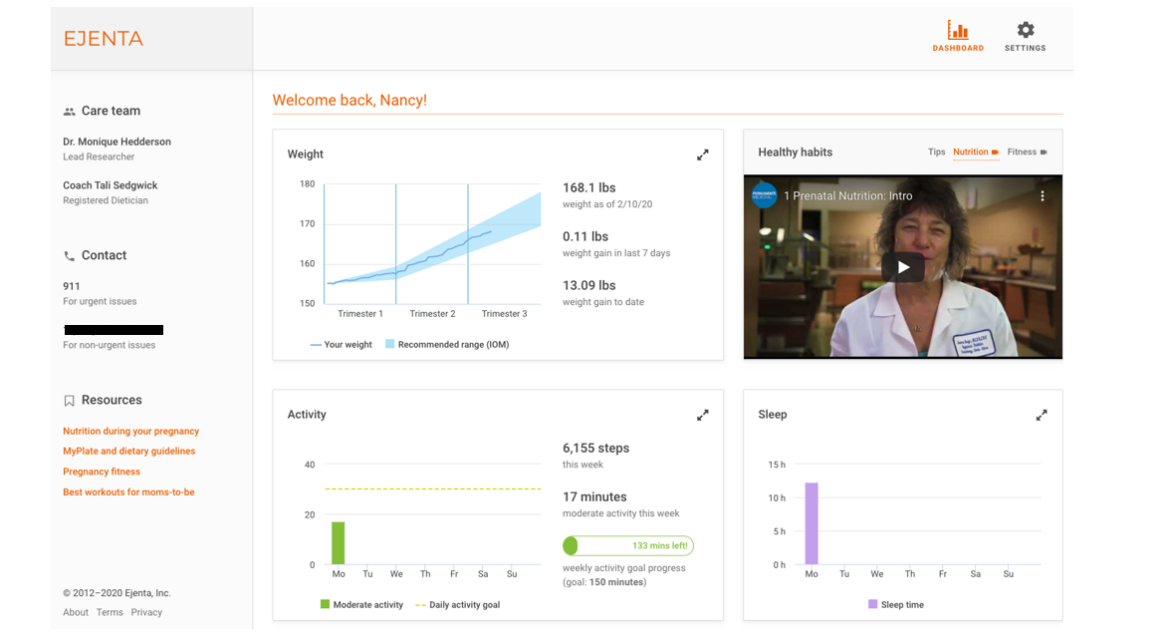
Representative screenshot of the Study of a Randomized Intervention Designed to Increase Exercise in Pregnancy (STRIDE) mobile health website.

The components of the intervention were as follows:

1. *Goal setting and check-in calls*—a one-time baseline visit conducted by a research assistant was held at the participant’s residence. During this visit, participants were oriented to the intervention and its tools and were asked to set a baseline goal for how many minutes of MVPA they would complete during the following 7 days. Participants were given print materials including a guidebook with 5 core sessions on the following topics: (1) Welcome to the STRIDE Program, (2) Getting Started with Physical Activity, (3) Getting Started with Healthy Eating, (4) Exercise Your Options, and (5) Talk Back Negative Thoughts. A lifestyle coach, a registered dietitian nutritionist with training in motivational interviewing, performed a check-in call 1 week later to help participants evaluate progress toward their goal and set a new activity goal for the following week. Subsequently, check-in calls were conducted monthly until the end of the pregnancy.
2. *Web-based, mHealth website*—the mHealth website was developed by the study team and engineers at the technology partner Ejenta, Inc. After the website was developed, it was beta tested with 5 pregnant patients to assess usability and understanding of the website’s components. The mHealth website was accessible via iPhone or Android smartphone or desktop through a unique log-in credential for each study participant. Real-time data from the activity tracker and scale were transmitted to the mHealth website. The website included a graph of participants’ GWG in relation to IOM guidelines and minutes of MVPA in relation to their goals (Figure 2) and pregnancy-related resources to help participants manage their GWG. A clinician portal enabled lifestyle coaches to view the participants’ self-monitoring data to tailor calls.
3. *Messages with personalized feedback*—participants received messages via email or SMS text message based on their preferences. Message content included reminders for self-weighing and self-monitoring PA; milestones at each trimester; and progress, goals, and milestones for PA (see samples in Textbox 1). Goal-achieved messages were sent whenever a participant reached a goal. Adherence reminder messages were sent the day the participant did not have weight or activity data (separate messages for weight vs activity). Motivational messages were sent once a week to encourage participants to reach their weekly activity goal. Activity milestone (personal best weekly, personal best daily, doubled activity and meeting goal, and activity in a row) messages were sent at the end of the day on Sunday if any milestones were achieved.

**Types of messages sent to Study of a Randomized Intervention Designed to Increase Exercise in Pregnancy (STRIDE) intervention arm participants.**

**Activity goal progress**

- “You had 129 minutes of moderate activity from (Monday, 5/8) to (Friday, 5/12). You need 21 more moderate minutes to reach your goal by Sunday. Let’s do this!”

**Activity goal reached**

- “Congrats on reaching your weekly activity goal! You’ve already been active for 150 minutes this week.”

**Activity milestones**

- Personal best (daily)
  - “You have a new personal best 62 minutes of moderate activity on (Friday, 5/12). Congrats!”
- Doubled daily activity goal
  - “Wow. You got over double your activity goal of 30 moderate minutes with 68 minutes on (Thursday, 5/11). You rock! Keep up the good work!”
- Met daily activity goal >3 days in a row
  - “You met your daily activity goal for 3 days in a row from (Monday, 5/8) to (Wednesday, 5/10). There’s no stopping you now! Power on!”

### Survey Overview

Study surveys were sent via email and were administered on the web. Survey 1 was administered at baseline and survey 2 was administered at 33 to 36 gestational age (GA) weeks. The surveys covered the self-assessed domains of pregnancy history, sleep, current PA, social support, quality of life, advice from their obstetrician or gynecologist, and demographic information. For intervention participants, survey 2 also included intervention evaluation questions. Survey participants received a US $30 Amazon gift card after completing both survey 1 and survey 2.

### Intervention Evaluation Outcomes

To assess the acceptability and feasibility of this pilot intervention, we analyzed adherence to self-monitoring (eg, wireless scale and wearable tracker) data and conducted surveys and focus groups with intervention participants to explore their satisfaction and experiences with the intervention. In the survey, participants ranked each intervention component on a 4-point Likert scale from very helpful to not at all helpful. Participants also rated the intervention as a whole on a 4-point Likert scale from fair to excellent and responded to whether they would recommend the program to other pregnant women on a 3-point Likert scale from *probably not* to *definitely yes*. In the focus groups, moderator guide questions examined suggestions for intervention improvement, impediments to regular use of intervention components, and overall intervention likes and dislikes. Focus groups were moderated by a registered dietitian and lifestyle coach and were conducted via WebEx. The focus group participants received a US $50 Amazon gift card for their participation.

We set a feasibility cutoff for self-monitoring (eg, self-weighing and wearing a wearable tracker) at ≥5 days per week as feasible for the participants. We set an acceptability cutoff for the perceived usefulness of the intervention at ≥80% helpful or very helpful responses for intervention component responses and good or very good or excellent for overall intervention responses to survey questions as acceptable for participants. We analyzed the focus group data to better understand what worked well for participants and to obtain information on how to improve the intervention for the larger pragmatic trial.

### Exploratory Outcomes

Although this pilot study was not powered for clinical outcomes, in addition to examining intervention acceptability and feasibility, we also assessed the intervention efficacy for various exploratory outcomes. The primary exploratory outcome was PA, measured both as total activity and in metabolic equivalent of task (MET) for that activity. MET is a measure of the intensity of PA. MET hours per week (total, moderate, or vigorous sports or exercise or moderate sports or exercise) and in activity minutes per week (moderate sports exercise). PA was self-assessed using the Pregnancy Physical Activity Questionnaire (PPAQ) [44] in both intervention arms. The PPAQ is an accurate and reliable measure of PA during pregnancy [44]. Participants reported the time spent in various PAs in the 2 months before completing the study. PAs were assessed in 5 domains: household or caregiving (13 activities), occupational (5 activities), sports and exercise (12 activities), transportation (3 activities), and inactivity or sedentary behavior (3 activities). For every activity, the participants selected 1 of 6 categorical responses for the time spent in that activity. Categorical responses included none, <0.5 hours per day, 0.5 to almost 1 hour per day, 1 to almost 2 hours per day, 2 to almost 3 hours per day, and ≥3 hours per day. Energy expended for each activity was calculated by multiplying the midpoint of the duration category reported spent in the activity by the corresponding MET for that activity. MET values for walking and light to moderate intensity household tasks were based on field-based measurements of pregnant women [45]. MET values for all other activities were based on the Compendium of Physical Activities [46]. Total duration and energy expenditure was calculated overall (including light, moderate, and vigorous PA) and separately for each intensity of PA (sedentary: <1.5 METs; light: 1.5 to <3 METs; moderate: 3 to 6 METs; vigorous: >6 METs). PA duration and energy expenditure overall and by intensity categories were the outcomes of interest. All patient weights were clinically assessed during the prenatal visits. Total GWG was defined as the last measured weight within 3 weeks before delivery minus the first measured weight after conception and up to 13 weeks of GA. The rate of total GWG was defined as the total GWG divided by the difference in GA weeks between the first and last measured weights during pregnancy. We also assessed the following perinatal outcomes as potential adverse events by using EHR data: gestational diabetes mellitus, preterm birth, large-for-GA, small-for-GA, and cesarean section.

### Analysis

All quantitative statistical analyses were conducted with SAS (version 9.4; SAS Institute Inc) and performed according to randomized group assignment (intention to treat), which included all participants for whom PA survey data or pregnancy weight measured after randomization were available. Multiple linear regression was used to estimate the point estimates and CIs of the overall difference between the usual care and intervention groups in change in activity in MET hours per week, change in activity in minutes per week, and GWG. All analyses were adjusted for the variables used in the adaptive randomization procedure and prepregnancy weight for GWG outcomes and baseline PA for PA outcomes. Therefore, our adjusted model for change in PA was adjusted for PA at baseline survey, age, parity, prepregnancy BMI, race or ethnicity, and difference in GA weeks between the baseline survey and the second survey. The adjusted model for GWG was adjusted for age, parity, prepregnancy BMI, race or ethnicity, and difference in GA weeks between the last and first measured weight during pregnancy.

To analyze the intervention evaluation data, we summarized the responses to the survey questions using frequencies. The focus groups were audio recorded and transcribed. We conducted directed content analysis [47] to analyze the focus group data. A coding guide was developed a priori based on the study’s conceptual framework, intervention components, and interview guide. The focus group transcripts were read to derive additional codes by highlighting words from the text that appeared to capture key thoughts or concepts. Labels for codes were developed that reflected more than one key thought. The final coding guide included codes reflecting topics from the conceptual framework and interview guide, intervention components, and inductively identified de novo topics. Codes were applied to the entire data set. Matrices were used to visually represent the data and to facilitate analysis by organizing and reducing the data.

## Results

### Overview

**Figure 3:**
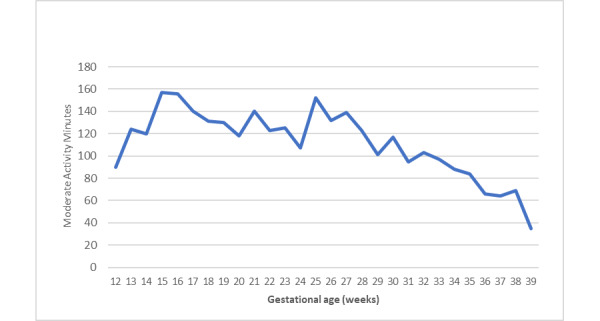
Mean moderate activity (minutes per week) by intervention group participants of the Study of a Randomized Intervention Designed to Increase Exercise in Pregnancy (STRIDE).

Among the 340 pregnant patients screened for eligibility, 46 (13.5%) were excluded owing to ineligibility and 204 (60%) declined or were unable to be reached. Among the 90 eligible patients, 75 (83%) consented to randomization. Following randomization, 89% (33/37) of the intervention participants and 92% (35/38) of the usual care participants completed all study surveys (*n*=35), and 89% (33/37) of the intervention participants and 84% (32/38) of the usual care participants had a weight measurement at the end of pregnancy (Figure 1). Reasons for loss to follow-up included pregnancy loss, change to insurance, and maximum contact attempts exceeded. The 2 study arms had similar baseline characteristics (Table 1). On average, participants in the intervention condition had a high adherence to self-monitoring: they wore the tracker 6 days per week and weighed themselves 5 times per week. In addition, in late pregnancy, 100% (33/33) of women in the intervention arm reported weighing themselves at least once a week compared with 20% (7/35) of women in the usual care arm. The intervention participants had an average of 112 mean minutes (SD 30 minutes) of moderate activity per week based on wearable tracker data across the entire intervention (Figure 3). On average, the mean minutes of moderate activity per week increased early in the intervention to a maximum of 157 minutes at 15 weeks of gestation and decreased during the third trimester to a minimum of 35 minutes at 39 weeks of gestation.

**Table 1:** Baseline characteristics by treatment condition: the Study of a Randomized Intervention Designed to Increase Exercise in Pregnancy (STRIDE) randomized controlled trial.

		Intervention (N=33)	Usual care (N=35)	*P* value	
Age (years), mean (SD)		34.8 (4.2)	33.2 (3.7)	.11	
**Prepregnancy BMI (kg/m^2^), mean (SD)**		28.9 (2.5)	28.9 (2.6)		
	25.0 to 29.9, n (%)	24 (73)	26 (74)	.94	
	30.0 to 40.0, n (%)	9 (27)	9 (26)	.88	
**Race-ethnicity, n (%)**					.82
	Asian	5 (15)	5 (14)		
	White	18 (55)	22 (63)		
	Hispanic	3 (9)	4 (11)		
	African American	1 (3)	1 (3)		
	Multiracial or other	6 (18)	3 (9)		
**Parity, n (%)**					.47
	0	20 (61)	17 (49)		
	1	12 (36)	15 (43)		
	>2	1 (3)	3 (9)		
**Household income per year (US $), n (%)**					.42
	**<**100,000	4 (12)	7 (20)		
	100,000 to 199,999	16 (49)	19 (54)		
	≥200,000	13 (39)	9 (26)		
**Education, n (%)**					.61
	High school or some college	3 (9)	6 (17)		
	College graduate (4-year course)	12 (36)	11 (31)		
	Postgraduate degree	18 (55)	18 (51)		
Gestational week at survey 1, mean (SD)		11.0 (1.8)	10.7 (1.3)	.36	
Gestational week at survey 2, mean (SD)		33.6 (1.0)	33.2 (0.4)	.04	

### Intervention Acceptability

We conducted an evaluation survey and 3 focus groups to better understand participants’ experiences with the intervention. A total of 33 participants in the intervention arm completed the evaluation survey (89% response rate). Overall, the mHealth lifestyle intervention was rated highly, with 88% (29/33) of the participants rating the intervention as excellent, very good, or good, and 85% (28/33) of the participants reporting that they would recommend the intervention to other pregnant patients.

A total of 22 participants agreed to be contacted for focus groups and were invited to participate in the qualitative study via email. A total of 14 participants enrolled in the 3 focus groups (64% response rate). The focus groups lasted for 40 to 50 minutes. Table 2 lists the ratings of each intervention component and the illustrative quotes related to the component. The digital scale was rated most highly (32/33, 97% of the participants rated it as very or moderately helpful) among all the intervention components followed by the coach calls (26/33, 79% of the participants rated it as very or moderately helpful), PA tracker, and text messages (24/33, 73% of the participants rated it as very or moderately helpful), with the mHealth website rated lowest (19/33, 58% of the participants rated it as very or moderately helpful).

**Table 2:** Program evaluation and acceptability results.

Intervention component	Survey results (n=33); “On the basis of your experience, how helpful was (were)...” (very or moderately helpful), n (%)	Qualitative results (n=14); illustrative quotes
Physical activity tracker	24 (73)	“I wore the activity tracker every day and I thought it was really helpful in monitoring my activity.”
Mobile health website	19 (58)	“I think also the visualization of the [website] was just helpful to get a sense of just how, I guess statistically how my body was changing.” “I also felt like the website, the STRIDE website, it didn’t—wasn’t—it didn’t feel particularly mobile-friendly, and I looked at it on my phone.”
Coach calls	26 (79)	• “I thought the lifestyle coach was great, also. [...] She was awesome, very concrete, and actually gave me usable advice versus just preaching.”
Digital scale	32 (97)	• “I liked the scale, the wireless scale, and having that linked to my phone so that I had kind of that instant feedback.” “I liked that there was always [something] to keep me accountable, the scale to keep me accountable, you know”
Text messages	24 (73)	“I enjoyed the congratulatory texts; they just made you feel good. The reminders were nice, especially if it was a little bit more to go, then it would give me that extra spur to take a look or something like that.”

Overall, the intervention was well received, and focus group participants reported that the mHealth lifestyle intervention helped them in 2 main ways. First, it promoted accountability. Second, the intervention motivated them and provided tangible support toward their PA goals. Similarly, the most highly rated intervention components (ie, digital scale, coach calls, PA tracker, and text messages) were cited as the most useful mechanisms for both accountability and motivation. The mHealth website was viewed more as a place to see all activities tracked in one place but less as a mechanism toward promoting accountability or motivation toward goals. Participants reported enjoying the mHealth lifestyle intervention program and believed that it led to more PA and less GWG. For example, participants said the following:

I think just the act of daily weighing and just paying attention to steps, especially I knew I was going to be going to the gym and working out for pregnancy, but the counting of the steps and the daily weigh-ins really quantified and made me pay attention to what I was doing. And I definitely walked around more and took the stairs and other stuff than I would have otherwise

My first pregnancy, I gained a lot more weight than with this one, and I credit a lot of it due to the STRIDE study, just being mindful and encouraged to be healthier and more active, I gained much less, significantly. So I was really grateful to be a part of it and I’m really glad I got to do it.

These quotes highlight the value of STRIDE to participants and how the tools provided by the intervention facilitated self-monitoring and improved health behaviors, including PA.

The focus group participants also mentioned potential improvements for the STRIDE intervention. Many participants felt that more nutrition information would have helped them achieve their GWG goals better. Ultimately, the study recommended, but did not require, popular free mHealth apps and websites such as MyFitnessPal and Choose MyPlate. Although there was mixed feedback on the use of these additional tools, many participants wanted more guidance on nutrition that was tailored to their actual dietary habits. For example, participants said the following:

I felt like the nutrition piece was nice to have as part of the overall study but wasn’t really integral in what we were doing, even though [...] that actually is a big factor in your weight, generally speaking. [...] Like, I think just more support in that area, but I don’t know that that support had to be necessarily talking to someone more than once a month. But I felt like it was an afterthought of the study, because it wasn’t even built into the program that you would track your eating.

I think where it fell down for me was a little bit around the nutrition, and because we weren’t really tracking that as part of the study, it felt like it was nice to have. So that—the orientation for the individual was more around the, like, eating piece. But we weren’t really tracking that, like.

Participants also wanted the mobile website to be a smartphone app. Many participants stated that they primarily used their mobile phones to access websites. Therefore, having an app or a more mobile-friendly website would facilitate website use.

### Exploratory Analyses

Although this pilot study was not powered for clinical outcomes, in exploratory analyses, we found that participants in the intervention had greater change in total activity per week compared with that in usual care (Table 3). Participants in the intervention arm had a 23.46 MET hours greater change in self-reported total PA per week (95% CI 1.13 to 45.8) and a 247.2-minute greater change in moderate intensity PA per week (95% CI 36.2 to 530.6) in unadjusted models, but this effect was attenuated in adjusted models (change in total PA: 15.55 MET hours per week, 95% CI −6.32 to 37.42; change in moderate intensity PA: 199.6 minutes per week, 95% CI −43.7 to 442.9). We found no difference between arms in total GWG (mean difference 1.14 kg, 95% CI −0.71 to 3.00) or rate of GWG (mean difference 0.03 kg, 95% CI −0.02 to 0.09). In addition, we did not find, and were not powered to find, any significant differences between intervention and usual care study arms in any adverse perinatal outcomes (data not shown).

**Table 3:** Change in physical activity and gestational weight gain (GWG) by treatment condition.

				Intervention (N=33), mean (SD)		Usual care (N=35), mean (SD)		Unadjusted model, mean difference (95% CI)		Adjusted model^a^, mean difference (95% CI)
**Activity in** **metabolic equivalent of task** **hours per week**										
	**Total activity**							23.46 (1.13 to 45.80)		15.55 (−6.32 to 37.42)
		8- to 15-week gestation	105.3 (62.7)		135.8 (100.2)					
		33- to 36-week gestation	108.9 (62.0)		115.9 (81.3)					
		Change	3.6 (36.8)		−19.9 (53.0)					
	**Moderate activity**							13.56 (−2.19 to 29.30		11.00 (−3.15 to 25.15)
		8- to 15-week gestation	49.0 (35.6)		65.7 (62.5)					
		33- to 36-week gestation	52.7 (33.8)		55.9 (51.4)					
		Change	3.7 (23.4)		−9.8 (39.0)					
	**Vigorous activity**							0.25 (−2.10 to 2.60)		0.25 (−2.10 to 2.60)
		8- to 15-week gestation	3.1 (4.8)		4.1 (6.1)					
		33- to 36-week gestation	1.0 (1.5)		1.7 (3.7)					
		Change	−2.1 (4.7)		−2.4 (4.9)					
	**Sports and exercise**							2.96 (−3.20 to 9.12)		2.46 (−2.23 to 7.16)
		8- to 15-week gestation	15.0 (14.8)		17.3 (21.4)					
		33- to 36-week gestation	12.6 (11.2)		12.0 (4.3)					
		Change	−2.4 (12.6)		−5.3 (12.6)					
	**Moderate sports and exercise**							2.71 (−2.06 to 7.47)		2.30 (−1.68 to 6.28)
		8- to 15-week gestation	12.0 (12.2)		13.2 (17.3)					
		33- to 36-week gestation	11.7 (11.0)		10.3 (12.0)					
		Change	−0.3 (10.1)		−3.0 (9.5)					
**Activity in minutes per week**										
	**Moderate activity**							247.2 (−36.2 to 530.6)		199.6 (−43.7 to 442.9)
		8- to 15-week gestation	818.5 (631.2)		1113.4 (1121.6)					
		33- to 36-week gestation	888.3 (596.2)		936.1 (872.9)					
		Change	69.9 (393.9)		−177.4 (716.8)					
	**Sports and exercise**							43.67 (−31.5 to 118.9)		35.98 (−23.3 to 95.23)
		8- to 15-week gestation	194.2 (173.8)		222.8 (267.9)					
		33- to 36-week gestation	183.8 (148.1)		168.7 (179.2)					
		Change	−10.4 (152.0)		−54.1 (156.1)					
**GWG**										
	Total GWG (kg^b^)		12.7 (3.8)		12.1 (4.1)		0.61 (−1.35 to 2.57)		1.14 (−0.71 to 3.00)	
	Rate of total GWG (kg/week)		0.4 (0.1)		0.4 (0.1)		0.01 (−0.05 to 0.07)		0.03 (−0.02 to 0.09)	

^a^Adjusted model for change in physical activity adjusted for physical activity at baseline survey, age, parity, prepregnancy BMI, race or ethnicity, and difference in gestational age (GA) weeks between baseline survey and the second survey. Adjusted model for GWG adjusted for age, parity, prepregnancy BMI, race or ethnicity, and difference in GA weeks between the last and first measured weight during pregnancy.

^b^Total GWG was defined as the last measured weight within 3 weeks before delivery minus the first measured weight after conception and up to 13 weeks of GA. Rate of total GWG was defined as total GWG divided by difference in GA weeks between the first and last measured weight during pregnancy.

## Discussion

### Principal Findings

In this mixed methods acceptability and feasibility randomized controlled pilot trial, we found that an mHealth intervention for pregnant patients with overweight or obesity was feasible and acceptable for participants and successfully promoted weight and PA self-monitoring. There was a high level of adherence to self-monitoring of weight and PA among participants in the intervention arm. and overall, the participants rated the program highly. Focus groups found that participants desired more support related to nutrition and a more mobile-friendly app instead of an mHealth website. In exploratory analyses, we found that the mHealth lifestyle intervention increased minutes of PA per week compared with usual care, but there was no difference in GWG. mHealth interventions with more nutrition support are likely needed to effectively affect GWG. It is important to note that although there are modifiable lifestyle factors (eg, nutrition and PA) that contribute to obesity, it is recognized as a complex, chronic disease driven by biological, genetic, environmental, and socioeconomic factors. Therefore, despite engagement with effective lifestyle interventions, pregnancy weight gain may differ among individuals because of a variety of factors outside of the scope of interventions, which may result in null intervention findings.

### Comparison With Prior Work

The findings of this study contribute to a small but growing body of literature with mixed results on mHealth interventions to improve PA in pregnant patients with overweight or obesity. Various pilot studies assessing the use of PA trackers (eg, Fitbit) to increase PA among pregnant patients have found no or small overall increases in steps [48-50]. However, Ainscough et al [51] found that an mHealth intervention, delivered via a smartphone app and grounded in behavior change techniques, increased motivation to engage in exercise, self-reported total PA (MET minutes per week), and moderate intensity PA (minutes per week) compared with the control group [51]. This suggests that mHealth interventions to increase PA among pregnant patients may be more effective when using behavior change theories and techniques. Our findings also point to the need for more research to better understand how to maximize the effectiveness of mHealth interventions in this population.

Our study also provides some additional exploratory evidence on the effects of mHealth lifestyle interventions on GWG. Multiple effective components (ie, daily self-monitoring of weight, PA, nutrition, goal setting, feedback, reinforcement, and problem solving) are likely needed to improve outcomes in pregnant patients with overweight or obesity. The lack of nutrition focus and food self-monitoring in our intervention could have contributed to null GWG findings. In a systematic review and meta-analysis of 11 exclusively digital interventions to encourage PA, appropriate weight gain during pregnancy, and healthy eating among pregnant patients, researchers found no overall benefit of exclusively digital interventions on GWG. However, effective individual interventions had twice as many behavior change techniques from *feedback and monitoring* domains and *goals and planning* domains than ineffective interventions. Moreover, higher user engagement with key behavior change techniques had a positive association with greater intervention effectiveness. Overall, effective interventions used both more behavior change techniques and interactivity in the form of personalized feedback, prompts to remind participants to use behavior change techniques and messages of encouragement, similar to our intervention [52]. Another systematic review and meta-analysis of 21 randomized controlled trials assessing the effects of technology-supported interventions on GWG found that these interventions had small effects on GWG, energy intake, eating behaviors, and PA. However, technology-supported interventions that included tracking tools, daily monitoring using devices, and face-to-face sessions were associated with slightly larger effects, particularly for PA [53].

Taken together, our findings demonstrate opportunities to (1) leverage technology to facilitate adherence to self-monitoring via automated, real-time transmission of weight and PA self-monitoring data, including real-time feedback on GWG in relation to the IOM guidelines and (2) incorporate tailored feedback from health care professionals (a lifestyle coach).

### Limitations

Our study had several limitations. First, this was a small randomized controlled pilot trial to assess the acceptability and feasibility of the intervention. Therefore, our study was not powered to detect clinical outcomes. Second, we relied on self-reported measures of PA, although PPAQ is a validated self-report tool for assessing PA during pregnancy. Third, pregnant patients randomized to the usual care arm had higher levels of baseline PA than those randomized to the intervention arm; however, we adjusted for baseline differences in our analysis. Fourth, this study used BMI measurements, which on their own, have limitations. However, these were the data available in the EHRs, and BMI is still widely used clinically because of its ease of measurement. Finally, the participants were not masked to the study group, which could have led to biased reports in the intervention arm.

### Conclusions

Our study demonstrated that the use of mHealth technology to deliver a theory-based lifestyle intervention is acceptable for pregnant patients with overweight or obesity. One goal of this pilot trial was to inform the design of a larger randomized controlled trial. To this end, the study team is currently implementing a pragmatic cluster randomized clinical trial that expands the pilot intervention based on participant feedback to incorporate additional nutrition support via nutrition self-monitoring and implements an app-based version of the pilot mHealth website to support better use. More effective interventions with broader reach are needed to help pregnant patients with overweight or obesity increase their PA and meet the IOM guidelines for GWG.

## Appendix

Multimedia Appendix 1 CONSORT-eHEALTH checklist (V 1.6.1).

## Abbreviations

EHR: electronic health record
GA: gestational age
GLOW: Gestational Weight Gain and Optimal Wellness
GWG: gestational weight gain
IOM: Institute of Medicine
KPNC: Kaiser Permanente Northern California
MET: metabolic equivalent of task
mHealth: mobile health
MVPA: moderate to vigorous intensity physical activity
PA: physical activity
PPAQ: Pregnancy Physical Activity Questionnaire
STRIDE: Study of a Randomized Intervention Designed to Increase Exercise in Pregnancy
