# Supplementary material for: A Web-Based mHealth Intervention With Telephone Support to Increase Physical Activity Among Pregnant Patients With Overweight or Obesity: Feasibility Randomized Controlled Trial
Source: JMIR Form Res. 2022 Jun 22;6(6):e33929. doi: 10.2196/33929 (PMC9260523; doi:10.2196/33929)
Supplement: Multimedia Appendix 1 [file formative_v6i6e33929_app1.pdf]

CONSORT-EHEALTH (V 1.6.1) - Submission/Publication Form

The CONSORT-EHEALTH checklist is intended for authors of randomized trials evaluating web-based and Internet-based applications/interventions, including mobile interventions, electronic games (incl multiplayer games), social media, certain telehealth applications, and other interactive and/or networked electronic applications. Some of the items (e.g. all subitems under item 5 - description of the intervention) may also be applicable for other study designs.

The goal of the CONSORT EHEALTH checklist and guideline is to be
a) a guide for reporting for authors of RCTs,
b) to form a basis for appraisal of an ehealth trial (in terms of validity)

CONSORT-EHEALTH items/subitems are MANDATORY reporting items for studies published in the Journal of Medical Internet Research and other journals / scientific societies endorsing the checklist.

Items numbered 1., 2., 3., 4a., 4b etc are original CONSORT or CONSORT-NPT (non-pharmacologic treatment) items.
Items with Roman numerals (i., ii, iii, iv etc.) are CONSORT-EHEALTH extensions/clarifications.

As the CONSORT-EHEALTH checklist is still considered in a formative stage, we would ask that you also RATE ON A SCALE OF 1-5 how important/useful you feel each item is FOR THE PURPOSE OF THE CHECKLIST and reporting guideline (optional).

Mandatory reporting items are marked with a red *.
In the textboxes, either copy & paste the relevant sections from your manuscript into this form - please include any quotes from your manuscript in QUOTATION MARKS,
or answer directly by providing additional information not in the manuscript, or elaborating on why the item was not relevant for this study.

YOUR ANSWERS WILL BE PUBLISHED AS A SUPPLEMENTARY FILE TO YOUR PUBLICATION IN JMIR AND ARE CONSIDERED PART OF YOUR PUBLICATION (IF ACCEPTED).
Please fill in these questions diligently. Information will not be copyedited, so please use proper spelling and grammar, use correct capitalization, and avoid abbreviations.

DO NOT FORGET TO SAVE AS PDF _AND_ CLICK THE SUBMIT BUTTON SO YOUR ANSWERS ARE IN OUR DATABASE !!!

Citation Suggestion (if you append the pdf as Appendix we suggest to cite this paper in the caption):
Eysenbach G, CONSORT-EHEALTH Group
CONSORT-EHEALTH: Improving and Standardizing Evaluation Reports of Web-based and Mobile Health Interventions

J Med Internet Res 2011;13(4):e126
URL: http://www.jmir.org/2011/4/e126/
doi: 10.2196/jmir.1923
PMID: 22209829

tainayah@stanford.edu (not shared) Switch account

Draft saved

* Required

Your name *

First Last

Tainayah Thomas

Primary Affiliation (short), City, Country *

University of Toronto, Toronto, Canada

Stanford University, Stanford, United States

Your e-mail address *

abc@gmail.com

tainayah@stanford.edu

Title of your manuscript *

Provide the (draft) title of your manuscript.

A web-based mobile-health intervention with telephone support to increase physical activity among pregnant patients with overweight or obesity: A feasibility randomized controlled trial

Name of your App/Software/Intervention *

If there is a short and a long/alternate name, write the short name first and add the long name in brackets.

STRIDE [Study of a Randomized Intervention D

Evaluated Version (if any)

e.g. "V1", "Release 2017-03-01", "Version 2.0.27913"

Your answer

Language(s) *

What language is the intervention/app in? If multiple languages are available, separate by comma (e.g. "English, French")

English

URL of your Intervention Website or App

e.g. a direct link to the mobile app on app in appstore (itunes, Google Play), or URL of the website. If the intervention is a DVD or hardware, you can also link to an Amazon page.

Your answer

URL of an image/screenshot (optional)

Your answer

Accessibility *

Can an enduser access the intervention presently?

- ○ access is free and open
- ○ access only for special usergroups, not open
- ○ access is open to everyone, but requires payment/subscription/in-app purchases
- ◉ app/intervention no longer accessible
- ○ Other:

Primary Medical Indication/Disease/Condition *

e.g. "Stress", "Diabetes", or define the target group in brackets after the condition, e.g. "Autism (Parents of children with)", "Alzheimers (Informal Caregivers of)"

Obesity (pregnant patients)

Primary Outcomes measured in trial *

comma-separated list of primary outcomes reported in the trial

physical activity

Secondary/other outcomes

Are there any other outcomes the intervention is expected to affect?

Your answer

Recommended "Dose" *

What do the instructions for users say on how often the app should be used?

- (●) Approximately Daily
- ( ) Approximately Weekly
- ( ) Approximately Monthly
- ( ) Approximately Yearly
- ( ) "as needed"
- ( ) Other:

Approx. Percentage of Users (starters) still using the app as recommended after 3 months *

- (●) unknown / not evaluated
- ( ) 0-10%
- ( ) 11-20%
- ( ) 21-30%
- ( ) 31-40%
- ( ) 41-50%
- ( ) 51-60%
- ( ) 61-70%
- ( ) 71%-80%
- ( ) 81-90%
- ( ) 91-100%
- ( ) Other:

Overall, was the app/intervention effective? *

- ( ) yes: all primary outcomes were significantly better in intervention group vs control
- ( ) partly: SOME primary outcomes were significantly better in intervention group vs control
- (•) no statistically significant difference between control and intervention
- ( ) potentially harmful: control was significantly better than intervention in one or more outcomes
- ( ) inconclusive: more research is needed
- ( ) Other:

Article Preparation Status/Stage *

At which stage in your article preparation are you currently (at the time you fill in this form)

- ( ) not submitted yet - in early draft status
- ( ) not submitted yet - in late draft status, just before submission
- ( ) submitted to a journal but not reviewed yet
- (•) submitted to a journal and after receiving initial reviewer comments
- ( ) submitted to a journal and accepted, but not published yet
- ( ) published
- ( ) Other:

Journal *

If you already know where you will submit this paper (or if it is already submitted), please provide the journal name (if it is not JMIR, provide the journal name under "other")

- ○ not submitted yet / unclear where I will submit this
- ○ Journal of Medical Internet Research (JMIR)
- ○ JMIR mHealth and UHealth
- ○ JMIR Serious Games
- ○ JMIR Mental Health
- ○ JMIR Public Health
- ◉ JMIR Formative Research
- ○ Other JMIR sister journal
- ○ Other:

Is this a full powered effectiveness trial or a pilot/feasibility trial? *

- ◉ Pilot/feasibility
- ○ Fully powered

Manuscript tracking number *

If this is a JMIR submission, please provide the manuscript tracking number under "other" (The ms tracking number can be found in the submission acknowledgement email, or when you login as author in JMIR. If the paper is already published in JMIR, then the ms tracking number is the four-digit number at the end of the DOI, to be found at the bottom of each published article in JMIR)

- ○ no ms number (yet) / not (yet) submitted to / published in JMIR
- ◉ Other: JFR ms#33929

TITLE AND ABSTRACT

1a) TITLE: Identification as a randomized trial in the title

1a) Does your paper address CONSORT item 1a? *

I.e does the title contain the phrase "Randomized Controlled Trial"? (if not, explain the reason under "other")

- (selected) yes
- ( ) Other:

1a-i) Identify the mode of delivery in the title

Identify the mode of delivery. Preferably use “web-based” and/or “mobile” and/or “electronic game” in the title. Avoid ambiguous terms like “online”, “virtual”, “interactive”. Use “Internet-based” only if Intervention includes non-web-based Internet components (e.g. email), use “computer-based” or “electronic” only if offline products are used. Use “virtual” only in the context of “virtual reality” (3-D worlds). Use “online” only in the context of “online support groups”. Complement or substitute product names with broader terms for the class of products (such as “mobile” or “smart phone” instead of “iphone”), especially if the application runs on different platforms.

	1	2	3	4	5	
subitem not at all important	○	○	○	●	○	essential

Clear selection

Does your paper address subitem 1a-i? *

Copy and paste relevant sections from manuscript title (include quotes in quotation marks "like this" to indicate direct quotes from your manuscript), or elaborate on this item by providing additional information not in the ms, or briefly explain why the item is not applicable/relevant for your study

"A web-based mobile-health intervention with telephone support to increase physical activity among pregnant patients with overweight or obesity: A feasibility randomized controlled trial"

1a-ii) Non-web-based components or important co-interventions in title

Mention non-web-based components or important co-interventions in title, if any (e.g., “with telephone support”).

	1	2	3	4	5	
subitem not at all important	○	○	◉	○	○	essential

Clear selection

Does your paper address subitem 1a-ii?

Copy and paste relevant sections from manuscript title (include quotes in quotation marks "like this" to indicate direct quotes from your manuscript), or elaborate on this item by providing additional information not in the ms, or briefly explain why the item is not applicable/relevant for your study

"A web-based mobile-health intervention with telephone support to increase physical activity among pregnant patients with overweight or obesity: A feasibility randomized controlled trial
"

1a-iii) Primary condition or target group in the title

Mention primary condition or target group in the title, if any (e.g., “for children with Type I Diabetes”)
Example: A Web-based and Mobile Intervention with Telephone Support for Children with Type I Diabetes: Randomized Controlled Trial

	1	2	3	4	5	
subitem not at all important	○	○	○	○	◉	essential

Clear selection

Does your paper address subitem 1a-iii? *

Copy and paste relevant sections from manuscript title (include quotes in quotation marks "like this" to indicate direct quotes from your manuscript), or elaborate on this item by providing additional information not in the ms, or briefly explain why the item is not applicable/relevant for your study

"A web-based mobile-health intervention with telephone support to increase physical activity among pregnant patients with overweight or obesity: A feasibility randomized controlled trial "

1b) ABSTRACT: Structured summary of trial design, methods, results, and conclusions

NPT extension: Description of experimental treatment, comparator, care providers, centers, and blinding status.

1b-i) Key features/functionalities/components of the intervention and comparator in the METHODS section of the ABSTRACT

Mention key features/functionalities/components of the intervention and comparator in the abstract. If possible, also mention theories and principles used for designing the site. Keep in mind the needs of systematic reviewers and indexers by including important synonyms. (Note: Only report in the abstract what the main paper is reporting. If this information is missing from the main body of text, consider adding it)

	1	2	3	4	5	
subitem not at all important	○	○	○	○	◉	essential

Clear selection

Does your paper address subitem 1b-i? *

Copy and paste relevant sections from the manuscript abstract (include quotes in quotation marks "like this" to indicate direct quotes from your manuscript), or elaborate on this item by providing additional information not in the ms, or briefly explain why the item is not applicable/relevant for your study

"Patients with singletons at 8–15 weeks' gestation, and aged 21 years or older with Wi-Fi access were recruited via email from two clinics within Kaiser Permanente Northern California and randomized to receive usual prenatal care or an mHealth lifestyle intervention. Participants in the intervention arm received wireless scales, access to an intervention website, activity trackers to receive automated feedback on weight gain and activity goals, and a monthly call from a lifestyle coach. Surveys and focus groups with intervention participants assessed intervention satisfaction and ways to improve the intervention. Physical activity outcomes were self-assessed using the Pregnancy Physical Activity Questionnaire and gestational weight gain was assessed using electronic health record data in both arms. "

1b-ii) Level of human involvement in the METHODS section of the ABSTRACT

Clarify the level of human involvement in the abstract, e.g., use phrases like "fully automated" vs. "therapist/nurse/care provider/physician-assisted" (mention number and expertise of providers involved, if any). (Note: Only report in the abstract what the main paper is reporting. If this information is missing from the main body of text, consider adding it)

	1	2	3	4	5	
subitem not at all important	○	○	●	○	○	essential

Clear selection

Does your paper address subitem 1b-ii?

Copy and paste relevant sections from the manuscript abstract (include quotes in quotation marks "like this" to indicate direct quotes from your manuscript), or elaborate on this item by providing additional information not in the ms, or briefly explain why the item is not applicable/relevant for your study

"monthly call from a lifestyle coach"

1b-iii) Open vs. closed, web-based (self-assessment) vs. face-to-face assessments in the METHODS section of the ABSTRACT

Mention how participants were recruited (online vs. offline), e.g., from an open access website or from a clinic or a closed online user group (closed usergroup trial), and clarify if this was a purely web-based trial, or there were face-to-face components (as part of the intervention or for assessment). Clearly say if outcomes were self-assessed through questionnaires (as common in web-based trials). Note: In traditional offline trials, an open trial (open-label trial) is a type of clinical trial in which both the researchers and participants know which treatment is being administered. To avoid confusion, use “blinded” or “unblinded” to indicated the level of blinding instead of “open”, as “open” in web-based trials usually refers to “open access” (i.e. participants can self-enrol). (Note: Only report in the abstract what the main paper is reporting. If this information is missing from the main body of text, consider adding it)

	1	2	3	4	5	
subitem not at all important	○	○	◉	○	○	essential

Clear selection

Does your paper address subitem 1b-iii?

Copy and paste relevant sections from the manuscript abstract (include quotes in quotation marks "like this" to indicate direct quotes from your manuscript), or elaborate on this item by providing additional information not in the ms, or briefly explain why the item is not applicable/relevant for your study

"Patients with singletons at 8–15 weeks’ gestation, and aged 21 years or older with Wi-Fi access were recruited via email from two clinics within Kaiser Permanente Northern California and randomized to receive usual prenatal care or an mHealth lifestyle intervention. Participants in the intervention arm received wireless scales, access to an intervention website, activity trackers to receive automated feedback on weight gain and activity goals, and a monthly call from a lifestyle coach. Surveys and focus groups with intervention participants assessed intervention satisfaction and ways to improve the intervention. Physical activity outcomes were self-assessed using the Pregnancy Physical Activity Questionnaire and gestational weight gain was assessed using electronic health record data in both arms. "

1b-iv) RESULTS section in abstract must contain use data

Report number of participants enrolled/assessed in each group, the use/uptake of the intervention (e.g., attrition/adherence metrics, use over time, number of logins etc.), in addition to primary/secondary outcomes. (Note: Only report in the abstract what the main paper is reporting. If this information is missing from the main body of text, consider adding it)

	1	2	3	4	5	
subitem not at all important	○	○	○	◉	○	essential

Clear selection

Does your paper address subitem 1b-iv?

Copy and paste relevant sections from the manuscript abstract (include quotes in quotation marks "like this" to indicate direct quotes from your manuscript), or elaborate on this item by providing additional information not in the ms, or briefly explain why the item is not applicable/relevant for your study

"Thirty-three patients were randomly assigned to the intervention arm and 35 patients to the usual care arm. 100% of participants in the intervention arm weighed themselves at least once a week compared with 20% of participants in the usual care arm. Participants in the intervention arm wore the activity tracker 6.4 days/week, weighed themselves 5.3 times/week, and 87.9% rated the program "good to excellent." Focus groups found participants desired more support related to nutrition to help them manage gestational weight gain and would have preferred an app instead of a website. Participants in the intervention arm had a 23.46 metabolic equivalent of task (MET)-hours greater change in total physical activity per week and a 247.2-minute greater change in moderate intensity physical activity per week in unadjusted models, but these effects were attenuated in adjusted models (change in total physical activity: 15.55 MET-hours per week; change in moderate intensity physical activity: 199.6 minutes per week. We found no difference in total gestational weight gain (mean difference: 1.14 kg compared to

1b-v) CONCLUSIONS/DISCUSSION in abstract for negative trials

Conclusions/Discussions in abstract for negative trials: Discuss the primary outcome - if the trial is negative (primary outcome not changed), and the intervention was not used, discuss whether negative results are attributable to lack of uptake and discuss reasons. (Note: Only report in the abstract what the main paper is reporting. If this information is missing from the main body of text, consider adding it)

	1	2	3	4	5	
subitem not at all important	○	◉	○	○	○	essential

Clear selection

Does your paper address subitem 1b-v?

Copy and paste relevant sections from the manuscript abstract (include quotes in quotation marks "like this" to indicate direct quotes from your manuscript), or elaborate on this item by providing additional information not in the ms, or briefly explain why the item is not applicable/relevant for your study

No, since our intervention was a pilot, feasibility study, we focus our conclusion on the feasibility aspect.

INTRODUCTION

2a) In INTRODUCTION: Scientific background and explanation of rationale

2a-i) Problem and the type of system/solution

Describe the problem and the type of system/solution that is object of the study: intended as stand-alone intervention vs. incorporated in broader health care program? Intended for a particular patient population? Goals of the intervention, e.g., being more cost-effective to other interventions, replace or complement other solutions? (Note: Details about the intervention are provided in "Methods" under 5)

	1	2	3	4	5	
subitem not at all important	○	○	◉	○	○	essential

Clear selection

Does your paper address subitem 2a-i? *

Copy and paste relevant sections from the manuscript (include quotes in quotation marks "like this" to indicate direct quotes from your manuscript), or elaborate on this item by providing additional information not in the ms, or briefly explain why the item is not applicable/relevant for your study

"Usual prenatal care includes regular weight measurements at prenatal care visits but during early pregnancy, these visits are infrequent, and patients may gain more than the Institute of Medicine (IOM) gestational weight gain guidelines between visits. This makes it critical to assess the impact of promoting self-weighing between visits and sharing this information with the patients' clinical care team to better evaluate and support guideline concordant weight gain." "This pilot acceptability and feasibility randomized controlled trial was developed to inform the design of a larger pragmatic cluster randomized controlled trial. The objective of this mixed-method study was to test the acceptability and feasibility of a pilot mHealth lifestyle intervention for pregnant patients with overweight or obesity to promote self-monitoring of weight and physical activity and encourage guideline-concordant gestational weight gain. "

2a-ii) Scientific background, rationale: What is known about the (type of) system

Scientific background, rationale: What is known about the (type of) system that is the object of the study (be sure to discuss the use of similar systems for other conditions/diagnoses, if appropiate), motivation for the study, i.e. what are the reasons for and what is the context for this specific study, from which stakeholder viewpoint is the study performed, potential impact of findings [2]. Briefly justify the choice of the comparator.

	1	2	3	4	5	
subitem not at all important	○	○	○	●	○	essential

Clear selection

Does your paper address subitem 2a-ii? *

Copy and paste relevant sections from the manuscript (include quotes in quotation marks "like this" to indicate direct quotes from your manuscript), or elaborate on this item by providing additional information not in the ms, or briefly explain why the item is not applicable/relevant for your study

"Current guidelines recommend pregnant patients get ≥ 30 minutes/day of moderate intensity physical activity (MVPA), most days of the week [16], but that goal is rarely achieved [17]. Therefore, interventions to improve physical activity (PA), especially among pregnant patients with overweight and obesity are needed. Commercially available technologies such as activity trackers (shown to increase PA in non-pregnant adults [18]) and wireless scales enable real-time self-monitoring, goal setting and tailored feedback on goals. Tailored feedback has been successful in reinforcing motivation for behavior change, especially when delivered in relation to goal attainment [19-21]. Utilizing technology to facilitate behavior change has the advantage of providing a resource that patients can use at their convenience without disrupting their busy lives. In addition, self-monitoring of weight with wireless scales can transmit weight data directly to health coaches and clinicians, allows for the objective measurement of daily weight, increases adherence compared with paper monitoring [22], and promotes weight loss in adults with overweight BMI, when used in conjunction with additional behavior change techniques [23]. While wireless scales and activity trackers may facilitate self-monitoring, provide just-in-time feedback and motivate change, few studies have both quantitatively and qualitatively evaluated the feasibility, acceptability, and efficacy of technology-based mHealth lifestyle interventions among pregnant patients with

2b) In INTRODUCTION: Specific objectives or hypotheses

Does your paper address CONSORT subitem 2b? *

Copy and paste relevant sections from the manuscript (include quotes in quotation marks "like this" to indicate direct quotes from your manuscript), or elaborate on this item by providing additional information not in the ms, or briefly explain why the item is not applicable/relevant for your study

"The objective of this mixed-method study was to test the acceptability and feasibility of a pilot mHealth lifestyle intervention for pregnant patients with overweight or obesity to promote self-monitoring of weight and physical activity and encourage guideline-concordant gestational weight gain. "

METHODS

3a) Description of trial design (such as parallel, factorial) including allocation ratio

Does your paper address CONSORT subitem 3a? *

Copy and paste relevant sections from the manuscript (include quotes in quotation marks "like this" to indicate direct quotes from your manuscript), or elaborate on this item by providing additional information not in the ms, or briefly explain why the item is not applicable/relevant for your study

"STRIDE (“Study of a Randomized Intervention Designed to Increase Exercise in Pregnancy”) is a two-arm, parallel group pilot randomized controlled trial "

3b) Important changes to methods after trial commencement (such as eligibility criteria), with reasons

Does your paper address CONSORT subitem 3b? *

Copy and paste relevant sections from the manuscript (include quotes in quotation marks "like this" to indicate direct quotes from your manuscript), or elaborate on this item by providing additional information not in the ms, or briefly explain why the item is not applicable/relevant for your study

We did not make changes to the methods after trial commencement.

3b-i) Bug fixes, Downtimes, Content Changes

Bug fixes, Downtimes, Content Changes: ehealth systems are often dynamic systems. A description of changes to methods therefore also includes important changes made on the intervention or comparator during the trial (e.g., major bug fixes or changes in the functionality or content) (5-iii) and other "unexpected events" that may have influenced study design such as staff changes, system failures/downtimes, etc. [2].

	1	2	3	4	5	
subitem not at all important	○	○	◉	○	○	essential

Clear selection

Does your paper address subitem 3b-i?

Copy and paste relevant sections from the manuscript (include quotes in quotation marks "like this" to indicate direct quotes from your manuscript), or elaborate on this item by providing additional information not in the ms, or briefly explain why the item is not applicable/relevant for your study

Your answer

4a) Eligibility criteria for participants

Does your paper address CONSORT subitem 4a? *

Copy and paste relevant sections from the manuscript (include quotes in quotation marks "like this" to indicate direct quotes from your manuscript), or elaborate on this item by providing additional information not in the ms, or briefly explain why the item is not applicable/relevant for your study

"Pregnant patients who were <12 gestational weeks and receiving care at KPNC's medical centers were first identified in the electronic health record (EHR). Eligibility criteria included: 1) age ≥ 21 years; 2) pre-pregnancy BMI between 25.0 and 40 kg/m2 (based on weight measured in the clinical setting within 12 months before the last menstrual period, or if unavailable, the first weight measured within the first 10 weeks of pregnancy); and 3) a singleton pregnancy. Eligibility was further assessed through a tiered process beginning with approval from medical providers to contact each patient and EHR review. Medical exclusion criteria that may impact outcome assessment, evaluated by the EHR review and by interview during a recruitment screening call, included multiple gestation; pregnancy loss; high-risk pregnancy (i.e., drug or alcohol abuse, chronic health problems, or pregnancy complications); thyroid disease diagnosed in the last 30 days; and use of glucose lowering medications or corticosteroids. Exclusion criteria that may interfere with full participation in the trial were assessed starting at the recruitment call and included plans to move out of the area or change health plan membership during pregnancy; no reliable access to a smartphone and Wi-Fi at home; unable to communicate in English; and not being willing to be randomized."

4a-i) Computer / Internet literacy

Computer / Internet literacy is often an implicit "de facto" eligibility criterion - this should be explicitly clarified.

	1	2	3	4	5	
subitem not at all important	○	◉	○	○	○	essential

Clear selection

Does your paper address subitem 4a-i?

Copy and paste relevant sections from the manuscript (include quotes in quotation marks "like this" to indicate direct quotes from your manuscript), or elaborate on this item by providing additional information not in the ms, or briefly explain why the item is not applicable/relevant for your study

Your answer

4a-ii) Open vs. closed, web-based vs. face-to-face assessments:

Open vs. closed, web-based vs. face-to-face assessments: Mention how participants were recruited (online vs. offline), e.g., from an open access website or from a clinic, and clarify if this was a purely web-based trial, or there were face-to-face components (as part of the intervention or for assessment), i.e., to what degree got the study team to know the participant. In online-only trials, clarify if participants were quasi-anonymous and whether having multiple identities was possible or whether technical or logistical measures (e.g., cookies, email confirmation, phone calls) were used to detect/prevent these.

	1	2	3	4	5	
subitem not at all important	○	○	◉	○	○	essential

Clear selection

Does your paper address subitem 4a-ii? *

Copy and paste relevant sections from the manuscript (include quotes in quotation marks "like this" to indicate direct quotes from your manuscript), or elaborate on this item by providing additional information not in the ms, or briefly explain why the item is not applicable/relevant for your study

"Patients were randomly assigned to the mHealth lifestyle intervention arm or the usual care control arm upon completion of a consent form and Survey 1 (Figure 1). The adaptive randomization procedure ensured that equivalent numbers of patients were assigned to each study arm and that the two study arms remained balanced overall and on each level of key characteristics: age (21 – 29.9, 30 – 34.9, and ≥ 35 years), pre-pregnancy BMI (25 – 29.9, 30 – 34.9, and 35 – 39.9 kg/m2), and race/ethnicity (Asian/Pacific Islander, Black, Hispanic, White, and multiethnic/other/unknown). Study participants were recruited via email with a follow-up phone call one week later. "

4a-iii) Information giving during recruitment

Information given during recruitment. Specify how participants were briefed for recruitment and in the informed consent procedures (e.g., publish the informed consent documentation as appendix, see also item X26), as this information may have an effect on user self-selection, user expectation and may also bias results.

	1	2	3	4	5	
subitem not at all important	○	◉	○	○	○	essential

Clear selection

Does your paper address subitem 4a-iii?

Copy and paste relevant sections from the manuscript (include quotes in quotation marks "like this" to indicate direct quotes from your manuscript), or elaborate on this item by providing additional information not in the ms, or briefly explain why the item is not applicable/relevant for your study

Your answer

4b) Settings and locations where the data were collected

Does your paper address CONSORT subitem 4b? *

Copy and paste relevant sections from the manuscript (include quotes in quotation marks "like this" to indicate direct quotes from your manuscript), or elaborate on this item by providing additional information not in the ms, or briefly explain why the item is not applicable/relevant for your study

"sample of pregnant patients with overweight or obesity from two Kaiser Permanente Northern California (KPNC) medical clinics."

4b-i) Report if outcomes were (self-)assessed through online questionnaires

Clearly report if outcomes were (self-)assessed through online questionnaires (as common in web-based trials) or otherwise.

	1	2	3	4	5	
subitem not at all important	○	○	●	○	○	essential

Clear selection

Does your paper address subitem 4b-i? *

Copy and paste relevant sections from the manuscript (include quotes in quotation marks "like this" to indicate direct quotes from your manuscript), or elaborate on this item by providing additional information not in the ms, or briefly explain why the item is not applicable/relevant for your study

"Study surveys were sent via email and administered online. Survey 1 was administered at baseline and survey 2 was administered at 33 to 36 gestational age (GA) weeks. The surveys covered self-assessed domains of pregnancy history, sleep, current physical activity, social support, quality of life, advice from their obstetrician/gynecologist, and demographic information. For intervention participants, survey 2 also included intervention evaluation questions." "While this pilot study was not powered for clinical outcomes, in addition to examining intervention acceptability and feasibility, we also assessed intervention efficacy for various exploratory outcomes. The primary exploratory outcome was physical activity, measured both as total activity and in metabolic equivalent of task (MET) for that activity. MET is a measure of intensity of physical activity. MET hours/week (total, moderate, vigorous, sports/exercise, moderate sports/exercise) and in activity minutes/week (moderate, sports exercise). Physical activity was self-assessed by the Pregnancy Physical Activity Questionnaire (PPAQ) [40] in both intervention arms."

4b-ii) Report how institutional affiliations are displayed

Report how institutional affiliations are displayed to potential participants [on ehealth media], as affiliations with prestigious hospitals or universities may affect volunteer rates, use, and reactions with regards to an intervention.(Not a required item – describe only if this may bias results)

	1	2	3	4	5	
subitem not at all important	◉	○	○	○	○	essential

Clear selection

Does your paper address subitem 4b-ii?

Copy and paste relevant sections from the manuscript (include quotes in quotation marks "like this" to indicate direct quotes from your manuscript), or elaborate on this item by providing additional information not in the ms, or briefly explain why the item is not applicable/relevant for your study

Your answer

5) The interventions for each group with sufficient details to allow replication, including how and when they were actually administered

5-i) Mention names, credential, affiliations of the developers, sponsors, and owners

Mention names, credential, affiliations of the developers, sponsors, and owners [6] (if authors/evaluators are owners or developer of the software, this needs to be declared in a “Conflict of interest” section or mentioned elsewhere in the manuscript).

	1	2	3	4	5	
subitem not at all important	○	○	●	○	○	essential

Clear selection

Does your paper address subitem 5-i?

Copy and paste relevant sections from the manuscript (include quotes in quotation marks "like this" to indicate direct quotes from your manuscript), or elaborate on this item by providing additional information not in the ms, or briefly explain why the item is not applicable/relevant for your study

"The mHealth site was developed by the study team and engineers at technology partner Ejenta, Inc. "

5-ii) Describe the history/development process

Describe the history/development process of the application and previous formative evaluations (e.g., focus groups, usability testing), as these will have an impact on adoption/use rates and help with interpreting results.

	1	2	3	4	5	
subitem not at all important	○	○	●	○	○	essential

Clear selection

Does your paper address subitem 5-ii?

Copy and paste relevant sections from the manuscript (include quotes in quotation marks "like this" to indicate direct quotes from your manuscript), or elaborate on this item by providing additional information not in the ms, or briefly explain why the item is not applicable/relevant for your study

"The mHealth site was developed by the study team and engineers at technology partner Ejenta, Inc. After the website was developed, it was beta tested with 5 pregnant patients to assess useability and understanding of the website's components"

5-iii) Revisions and updating

Revisions and updating. Clearly mention the date and/or version number of the application/intervention (and comparator, if applicable) evaluated, or describe whether the intervention underwent major changes during the evaluation process, or whether the development and/or content was "frozen" during the trial. Describe dynamic components such as news feeds or changing content which may have an impact on the replicability of the intervention (for unexpected events see item 3b).

	1	2	3	4	5	
subitem not at all important	○	○	◉	○	○	essential

Clear selection

Does your paper address subitem 5-iii?

Copy and paste relevant sections from the manuscript (include quotes in quotation marks "like this" to indicate direct quotes from your manuscript), or elaborate on this item by providing additional information not in the ms, or briefly explain why the item is not applicable/relevant for your study

Our website/intervention did not have any major revisions or updates

5-iv) Quality assurance methods

Provide information on quality assurance methods to ensure accuracy and quality of information provided [1], if applicable.

	1	2	3	4	5	
subitem not at all important	○	◉	○	○	○	essential

Clear selection

Does your paper address subitem 5-iv?

Copy and paste relevant sections from the manuscript (include quotes in quotation marks "like this" to indicate direct quotes from your manuscript), or elaborate on this item by providing additional information not in the ms, or briefly explain why the item is not applicable/relevant for your study

Your answer

5-v) Ensure replicability by publishing the source code, and/or providing screenshots/screen-capture video, and/or providing flowcharts of the algorithms used

Ensure replicability by publishing the source code, and/or providing screenshots/screen-capture video, and/or providing flowcharts of the algorithms used. Replicability (i.e., other researchers should in principle be able to replicate the study) is a hallmark of scientific reporting.

	1	2	3	4	5	
subitem not at all important	○	◉	○	○	○	essential

Clear selection

Does your paper address subitem 5-v?

Copy and paste relevant sections from the manuscript (include quotes in quotation marks "like this" to indicate direct quotes from your manuscript), or elaborate on this item by providing additional information not in the ms, or briefly explain why the item is not applicable/relevant for your study

We did include a screenshot of the study website as Figure 2

5-vi) Digital preservation

Digital preservation: Provide the URL of the application, but as the intervention is likely to change or disappear over the course of the years; also make sure the intervention is archived (Internet Archive, webcitation.org, and/or publishing the source code or screenshots/videos alongside the article). As pages behind login screens cannot be archived, consider creating demo pages which are accessible without login.

	1	2	3	4	5	
subitem not at all important	(selected)	○	○	○	○	essential

Clear selection

Does your paper address subitem 5-vi?

Copy and paste relevant sections from the manuscript (include quotes in quotation marks "like this" to indicate direct quotes from your manuscript), or elaborate on this item by providing additional information not in the ms, or briefly explain why the item is not applicable/relevant for your study

Your answer

5-vii) Access

Access: Describe how participants accessed the application, in what setting/context, if they had to pay (or were paid) or not, whether they had to be a member of specific group. If known, describe how participants obtained "access to the platform and Internet" [1]. To ensure access for editors/reviewers/readers, consider to provide a "backdoor" login account or demo mode for reviewers/readers to explore the application (also important for archiving purposes, see vi).

	1	2	3	4	5	
subitem not at all important	○	○	●	○	○	essential

Clear selection

Does your paper address subitem 5-vii? *

Copy and paste relevant sections from the manuscript (include quotes in quotation marks "like this" to indicate direct quotes from your manuscript), or elaborate on this item by providing additional information not in the ms, or briefly explain why the item is not applicable/relevant for your study

"The mHealth website was accessible via iPhone or Android smartphone, or desktop through a unique login credential for each study participant. Real-time data from the activity tracker and scale were transmitted to the mHealth site."

5-viii) Mode of delivery, features/functionalities/components of the intervention and comparator, and the theoretical framework

Describe mode of delivery, features/functionalities/components of the intervention and comparator, and the theoretical framework [6] used to design them (instructional strategy [1], behaviour change techniques, persuasive features, etc., see e.g., [7, 8] for terminology). This includes an in-depth description of the content (including where it is coming from and who developed it) [1]," whether [and how] it is tailored to individual circumstances and allows users to track their progress and receive feedback" [6]. This also includes a description of communication delivery channels and – if computer-mediated communication is a component – whether communication was synchronous or asynchronous [6]. It also includes information on presentation strategies [1], including page design principles, average amount of text on pages, presence of hyperlinks to other resources, etc. [1].

	1	2	3	4	5	
subitem not at all important	○	○	○	●	○	essential

Clear selection

Does your paper address subitem 5-viii? *

Copy and paste relevant sections from the manuscript (include quotes in quotation marks "like this" to indicate direct quotes from your manuscript), or elaborate on this item by providing additional information not in the ms, or briefly explain why the item is not applicable/relevant for your study

"Intervention components:
1. Goal setting and check-in calls – A one-time baseline visit conducted by a research assistant was held at the participant's residence. During this visit, participants were oriented to the intervention and its tools and were asked to set a baseline goal for how many minutes of MVPA they would complete during the following seven days. Participants were given print materials including a guidebook of 5 core sessions on the following topics: 1-Welcome to the STRIDE Program, 2-Getting Started with Physical Activity, 3-Getting Started with Healthy Eating, 4-Exercise Your Options, 5-Talk Back Negative Thoughts. A lifestyle coach, a registered dietitian nutritionist with training in motivational interviewing, performed a check-in call 1-week later to help participants evaluate progress toward their goal and set at new activity goal for the following week. Subsequently, check-in calls were conducted monthly until the end of pregnancy.
2. Web-based, Mobile health (mHealth) website – The mHealth site was developed by the study team and engineers at technology partner Ejenta, Inc. After the website was developed, it was beta tested with 5 pregnant patients to assess useability and understanding of the website's components. The mHealth website was accessible via iPhone or Android smartphone, or desktop through a unique login credential for each study participant. Real-time data from the activity tracker and scale were transmitted to the mHealth site. The site included a graph of participants' gestational weight gain in relation to IOM guidelines and minutes of MVPA in relation to their goals (Figure 2), and pregnancy-related resources to help participants manage their gestational weight gain. A clinician portal enabled lifestyle coaches to see participants' self-monitoring data to tailor calls.

3. Messages with personalized feedback – Participants received messages via email or text message based on their preference. Message content included reminders for self-weighing and self-monitoring physical activity; milestones at each trimester; and progress, goals, and milestones for physical activity (see samples in Table 1 below), Goal achieved messages were sent whenever a participant reached a goal. Adherence reminder messages were sent the day the participant did not have weight or activity data(separate messages for weight vs activity). (Separate messages for weight vs activity). Motivational messages were sent once a week to help encourage participants to reach weekly activity goal. Activity milestone (personal best weekly, personal best daily, doubled activity and meeting goal, activity in a row) messages were sent end of day on Sunday if any milestones were achieved. "

5-ix) Describe use parameters

Describe use parameters (e.g., intended “doses” and optimal timing for use). Clarify what instructions or recommendations were given to the user, e.g., regarding timing, frequency, heaviness of use, if any, or was the intervention used ad libitum.

	1	2	3	4	5	
subitem not at all important	○	○	●	○	○	essential

Clear selection

Does your paper address subitem 5-ix?

Copy and paste relevant sections from the manuscript (include quotes in quotation marks "like this" to indicate direct quotes from your manuscript), or elaborate on this item by providing additional information not in the ms, or briefly explain why the item is not applicable/relevant for your study

"Physical Activity: Participants in the intervention arm were asked to set physical activity goals and gradually increase their activity to ultimately reach 150 minutes of moderate-to-vigorous activity per week, in accordance with current American College of Obstetricians and Gynecologists (ACOG) recommendations [16]. Participants were provided with a Withings Activité Pop physical activity tracker that is worn on the wrist and tracks daily steps and minutes of moderate-to-vigorous intensity physical activity (MVPA) based on a 3-axis accelerometer optoelectronics sensor with intensity based on the metabolic coefficient ≥ 3. Withings activity trackers are among the most accurate at measuring steps and MVPA [36, 37], with the lowest rate of false positive steps [38]. Participants were encouraged to wear their tracker daily.
Self-weighing: Participants were provided with a Withings Body digital scale that transmits weights via Wi-Fi or Bluetooth and has margin of error on weight <200 g against a gold standard scale [39]. Participants were encouraged to weigh themselves at home daily. "

5-x) Clarify the level of human involvement

Clarify the level of human involvement (care providers or health professionals, also technical assistance) in the e-intervention or as co-intervention (detail number and expertise of professionals involved, if any, as well as “type of assistance offered, the timing and frequency of the support, how it is initiated, and the medium by which the assistance is delivered”. It may be necessary to distinguish between the level of human involvement required for the trial, and the level of human involvement required for a routine application outside of a RCT setting (discuss under item 21 – generalizability).

	1	2	3	4	5	
subitem not at all important	○	○	◉	○	○	essential

Clear selection

Does your paper address subitem 5-x?

Copy and paste relevant sections from the manuscript (include quotes in quotation marks "like this" to indicate direct quotes from your manuscript), or elaborate on this item by providing additional information not in the ms, or briefly explain why the item is not applicable/relevant for your study

"1. Goal setting and check-in calls – A one-time baseline visit conducted by a research assistant was held at the participant’s residence. During this visit, participants were oriented to the intervention and its tools and were asked to set a baseline goal for how many minutes of MVPA they would complete during the following seven days. Participants were given print materials including a guidebook of 5 core sessions on the following topics: 1-Welcome to the STRIDE Program, 2-Getting Started with Physical Activity, 3-Getting Started with Healthy Eating, 4-Exercise Your Options, 5-Talk Back Negative Thoughts. A lifestyle coach, a registered dietitian nutritionist with training in motivational interviewing, performed a check-in call 1-week later to help participants evaluate progress toward their goal and set at new activity goal for the following week. Subsequently, check-in calls were conducted monthly until the end of pregnancy. "

5-xi) Report any prompts/reminders used

Report any prompts/reminders used: Clarify if there were prompts (letters, emails, phone calls, SMS) to use the application, what triggered them, frequency etc. It may be necessary to distinguish between the level of prompts/reminders required for the trial, and the level of prompts/reminders for a routine application outside of a RCT setting (discuss under item 21 – generalizability).

	1	2	3	4	5	
subitem not at all important	○	○	○	◉	○	essential

Clear selection

Does your paper address subitem 5-xi? *

Copy and paste relevant sections from the manuscript (include quotes in quotation marks "like this" to indicate direct quotes from your manuscript), or elaborate on this item by providing additional information not in the ms, or briefly explain why the item is not applicable/relevant for your study

"Participants received messages via email or text message based on their preference. Message content included reminders for self-weighing and self-monitoring physical activity; milestones at each trimester; and progress, goals, and milestones for physical activity (see samples in Table 1 below), Goal achieved messages were sent whenever a participant reached a goal. Adherence reminder messages were sent the day the participant did not have weight or activity data(separate messages for weight vs activity). (Separate messages for weight vs activity). Motivational messages were sent once a week to help encourage participants to reach weekly activity goal. Activity milestone (personal best weekly, personal best daily, doubled activity and meeting goal, activity in a row) messages were sent end of day on Sunday if any milestones were

5-xii) Describe any co-interventions (incl. training/support)

Describe any co-interventions (incl. training/support): Clearly state any interventions that are provided in addition to the targeted eHealth intervention, as ehealth intervention may not be designed as stand-alone intervention. This includes training sessions and support [1]. It may be necessary to distinguish between the level of training required for the trial, and the level of training for a routine application outside of a RCT setting (discuss under item 21 – generalizability.

	1	2	3	4	5	
subitem not at all important	○	○	○	◉	○	essential

Clear selection

Does your paper address subitem 5-xii? *

Copy and paste relevant sections from the manuscript (include quotes in quotation marks "like this" to indicate direct quotes from your manuscript), or elaborate on this item by providing additional information not in the ms, or briefly explain why the item is not applicable/relevant for your study

There were no co-interventions/trainings provided.

6a) Completely defined pre-specified primary and secondary outcome measures, including how and when they were assessed

Does your paper address CONSORT subitem 6a? *

Copy and paste relevant sections from the manuscript (include quotes in quotation marks "like this" to indicate direct quotes from your manuscript), or elaborate on this item by providing additional information not in the ms, or briefly explain why the item is not applicable/relevant for your study

"To assess acceptability and feasibility of this pilot intervention, we analyzed adherence to self-monitoring (e.g., wireless scale, wearable tracker) data, and conducted surveys and focus groups with intervention participants to explore their satisfaction and experiences with the intervention. In the survey, participants ranked each intervention component on a four-point Likert scale from very helpful to not at all helpful. Participants also rated the intervention as whole on a four-point Likert scale from fair to excellent and responded to whether they would recommend the program to other pregnant women on a three-point Likert scale from probably not to definitely yes. In focus groups, moderator guide questions examined suggestions for intervention improvement, impediments to regular use of intervention components and overall intervention likes and dislikes. Focus groups were moderated by a registered dietitian and lifestyle coach and were conducted virtually via WebEx. Focus groups participants received a $50 Amazon gift card for their participation.
We set a feasibility cut-off for self-monitoring (e.g., self-weighing, wearing wearable tracker) at ≥5 days per week as feasible for participants. We set an acceptability cut-off for perceived usefulness of the intervention at ≥80% helpful/very helpful responses for intervention component responses and good/very good/ excellent for overall intervention responses to survey questions as acceptable for participants. We analyzed focused group data to better understand what worked well for participants and to obtain information on how to improve the intervention for the larger pragmatic trial.
Exploratory Outcomes
While this pilot study was not powered for clinical outcomes, in addition to examining intervention acceptability and feasibility, we also assessed intervention efficacy for various exploratory outcomes. The primary exploratory outcome was physical activity, measured both as total activity and in metabolic equivalent of task (MET) for that activity. MET is a measure of intensity of physical activity. MET hours/week (total, moderate, vigorous, sports/exercise, moderate sports/exercise) and in activity minutes/week (moderate, sports exercise). Physical activity was self-assessed by the Pregnancy Physical Activity Questionnaire (PPAQ) [40] in both intervention arms. "

6a-i) Online questionnaires: describe if they were validated for online use and apply CHERRIES items to describe how the questionnaires were designed/deployed

If outcomes were obtained through online questionnaires, describe if they were validated for online use and apply CHERRIES items to describe how the questionnaires were designed/deployed [9].

	1	2	3	4	5	
subitem not at all important	○	○	◉	○	○	essential

Clear selection

Does your paper address subitem 6a-i?

Copy and paste relevant sections from manuscript text

Your answer

6a-ii) Describe whether and how “use” (including intensity of use/dosage) was defined/measured/monitored

Describe whether and how “use” (including intensity of use/dosage) was defined/measured/monitored (logins, logfile analysis, etc.). Use/adoption metrics are important process outcomes that should be reported in any ehealth trial.

	1	2	3	4	5	
subitem not at all important	○	○	◉	○	○	essential

Clear selection

Does your paper address subitem 6a-ii?

Copy and paste relevant sections from manuscript text

"We set a feasibility cut-off for self-monitoring (e.g., self-weighing, wearing wearable tracker) at ≥5 days per week as feasible for participants."

6a-iii) Describe whether, how, and when qualitative feedback from participants was obtained

Describe whether, how, and when qualitative feedback from participants was obtained (e.g., through emails, feedback forms, interviews, focus groups).

	1	2	3	4	5	
subitem not at all important	○	○	◉	○	○	essential

Clear selection

Does your paper address subitem 6a-iii?

Copy and paste relevant sections from manuscript text

"In focus groups, moderator guide questions examined suggestions for intervention improvement, impediments to regular use of intervention components and overall intervention likes and dislikes. Focus groups were moderated by a registered dietitian and lifestyle coach and were conducted virtually via WebEx. "

6b) Any changes to trial outcomes after the trial commenced, with reasons

Does your paper address CONSORT subitem 6b? *

Copy and paste relevant sections from the manuscript (include quotes in quotation marks "like this" to indicate direct quotes from your manuscript), or elaborate on this item by providing additional information not in the ms, or briefly explain why the item is not applicable/relevant for your study

There were no changes to study outcomes after the trial commenced.

7a) How sample size was determined

NPT: When applicable, details of whether and how the clustering by care provides or centers was addressed

7a-i) Describe whether and how expected attrition was taken into account when calculating the sample size

Describe whether and how expected attrition was taken into account when calculating the sample size.

	1	2	3	4	5	
subitem not at all important	○	◉	○	○	○	essential

Clear selection

Does your paper address subitem 7a-i?

Copy and paste relevant sections from manuscript title (include quotes in quotation marks "like this" to indicate direct quotes from your manuscript), or elaborate on this item by providing additional information not in the ms, or briefly explain why the item is not applicable/relevant for your study

Your answer

7b) When applicable, explanation of any interim analyses and stopping guidelines

Does your paper address CONSORT subitem 7b? *

Copy and paste relevant sections from the manuscript (include quotes in quotation marks "like this" to indicate direct quotes from your manuscript), or elaborate on this item by providing additional information not in the ms, or briefly explain why the item is not applicable/relevant for your study

Not applicable

Your answer must have a minimum of 25 characters.

8a) Method used to generate the random allocation sequence

NPT: When applicable, how care providers were allocated to each trial group

Does your paper address CONSORT subitem 8a? *

Copy and paste relevant sections from the manuscript (include quotes in quotation marks "like this" to indicate direct quotes from your manuscript), or elaborate on this item by providing additional information not in the ms, or briefly explain why the item is not applicable/relevant for your study

"Patients were randomly assigned to the mHealth lifestyle intervention arm or the usual care control arm upon completion of a consent form and Survey 1 (Figure 1). The adaptive randomization procedure ensured that equivalent numbers of patients were assigned to each study arm and that the two study arms remained balanced overall and on each level of key characteristics: age (21 – 29.9, 30 – 34.9, and ≥ 35 years), pre-pregnancy BMI (25 – 29.9, 30 – 34.9, and 35 – 39.9 kg/m2), and race/ethnicity (Asian/Pacific Islander, Black, Hispanic, White, and multiethnic/other/unknown). "

8b) Type of randomisation; details of any restriction (such as blocking and block size)

Does your paper address CONSORT subitem 8b? *

Copy and paste relevant sections from the manuscript (include quotes in quotation marks "like this" to indicate direct quotes from your manuscript), or elaborate on this item by providing additional information not in the ms, or briefly explain why the item is not applicable/relevant for your study

"Patients were randomly assigned to the mHealth lifestyle intervention arm or the usual care control arm upon completion of a consent form and Survey 1 (Figure 1). The adaptive randomization procedure ensured that equivalent numbers of patients were assigned to each study arm and that the two study arms remained balanced overall and on each level of key characteristics: age (21 – 29.9, 30 – 34.9, and ≥ 35 years), pre-pregnancy BMI (25 – 29.9, 30 – 34.9, and 35 – 39.9 kg/m2), and race/ethnicity (Asian/Pacific Islander, Black, Hispanic, White, and multiethnic/other/unknown). "

9) Mechanism used to implement the random allocation sequence (such as sequentially numbered containers), describing any steps taken to conceal the sequence until interventions were assigned

Does your paper address CONSORT subitem 9? *

Copy and paste relevant sections from the manuscript (include quotes in quotation marks "like this" to indicate direct quotes from your manuscript), or elaborate on this item by providing additional information not in the ms, or briefly explain why the item is not applicable/relevant for your study

We did not take any specific steps to conceal the sequence

10) Who generated the random allocation sequence, who enrolled participants, and who assigned participants to interventions

Does your paper address CONSORT subitem 10? *

Copy and paste relevant sections from the manuscript (include quotes in quotation marks "like this" to indicate direct quotes from your manuscript), or elaborate on this item by providing additional information not in the ms, or briefly explain why the item is not applicable/relevant for your study

"Study participants were recruited via email by study staff with a follow-up phone call one week later. "

11a) If done, who was blinded after assignment to interventions (for example, participants, care providers, those assessing outcomes) and how

NPT: Whether or not administering co-interventions were blinded to group assignment

11a-i) Specify who was blinded, and who wasn't

Specify who was blinded, and who wasn't. Usually, in web-based trials it is not possible to blind the participants [1, 3] (this should be clearly acknowledged), but it may be possible to blind outcome assessors, those doing data analysis or those administering co-interventions (if any).

	1	2	3	4	5	
subitem not at all important	○	○	●	○	○	essential

Clear selection

Does your paper address subitem 11a-i? *

Copy and paste relevant sections from the manuscript (include quotes in quotation marks "like this" to indicate direct quotes from your manuscript), or elaborate on this item by providing additional information not in the ms, or briefly explain why the item is not applicable/relevant for your study

'The biostatistician, clinicians, research assistant who sent out study-related emails and surveys, and investigators were masked to study arm assignment."

11a-ii) Discuss e.g., whether participants knew which intervention was the “intervention of interest” and which one was the “comparator”

Informed consent procedures (4a-ii) can create biases and certain expectations - discuss e.g., whether participants knew which intervention was the “intervention of interest” and which one was the “comparator”.

	1	2	3	4	5	
subitem not at all important	○	◉	○	○	○	essential

Clear selection

Does your paper address subitem 11a-ii?

Copy and paste relevant sections from the manuscript (include quotes in quotation marks "like this" to indicate direct quotes from your manuscript), or elaborate on this item by providing additional information not in the ms, or briefly explain why the item is not applicable/relevant for your study

Your answer

11b) If relevant, description of the similarity of interventions

(this item is usually not relevant for ehealth trials as it refers to similarity of a placebo or sham intervention to a active medication/intervention)

Does your paper address CONSORT subitem 11b? *

Copy and paste relevant sections from the manuscript (include quotes in quotation marks "like this" to indicate direct quotes from your manuscript), or elaborate on this item by providing additional information not in the ms, or briefly explain why the item is not applicable/relevant for your study

"Usual care
Participants randomized to the usual care arm received standard KPNC prenatal medical care. This includes an initial prenatal visit at 7-10 weeks' gestation and a newsletter containing the Institute of Medicine (IOM) gestational weight gain guidelines and advice on healthy eating. Participants with routine pregnancies then received an additional 7 prenatal visits between 16 weeks' gestation and delivery. Medical staff weighed patients at each visit per standard care.
Intervention
In addition to usual care described above, patients randomized to the intervention arm received a multi-component mHealth lifestyle intervention. "

12a) Statistical methods used to compare groups for primary and secondary outcomes

NPT: When applicable, details of whether and how the clustering by care providers or centers was addressed

Does your paper address CONSORT subitem 12a? *

Copy and paste relevant sections from the manuscript (include quotes in quotation marks "like this" to indicate direct quotes from your manuscript), or elaborate on this item by providing additional information not in the ms, or briefly explain why the item is not applicable/relevant for your study

"All quantitative statistical analyses were conducted with SAS 9.4 and done according to randomized group assignment (intention-to-treat), which included all participants for whom physical activity survey data or pregnancy weight measured after randomization was available. Multiple linear regression was used to estimate point estimates and confidence intervals of the overall difference between usual care and intervention groups in change in activity in MET hours/week, change in activity in minutes per week, and GWG. All analyses were adjusted for the variables used in the adaptive randomization procedure as well as pre-pregnancy weight for gestational weight gain outcomes and baseline physical activity for physical activity outcomes. Therefore, our adjusted model for change in physical activity adjusted for physical activity at baseline survey, age, parity, pre-pregnancy BMI, race/ethnicity, and difference in GA weeks between baseline survey and the second survey. The adjusted model for GWG adjusted for age, parity, pre-pregnancy BMI, race/ethnicity, and difference in GA weeks between last and first measured weight during pregnancy.
To analyze intervention evaluation data, we summarized responses to survey questions using frequencies. "

12a-i) Imputation techniques to deal with attrition / missing values

Imputation techniques to deal with attrition / missing values: Not all participants will use the intervention/comparator as intended and attrition is typically high in ehealth trials. Specify how participants who did not use the application or dropped out from the trial were treated in the statistical analysis (a complete case analysis is strongly discouraged, and simple imputation techniques such as LOCF may also be problematic [4]).

	1	2	3	4	5	
subitem not at all important	○	○	●	○	○	essential

Clear selection

Does your paper address subitem 12a-i? *

Copy and paste relevant sections from the manuscript (include quotes in quotation marks "like this" to indicate direct quotes from your manuscript), or elaborate on this item by providing additional information not in the ms, or briefly explain why the item is not applicable/relevant for your study

"All quantitative statistical analyses were conducted with SAS 9.4 and done according to randomized group assignment (intention-to-treat), which included all participants for whom physical activity survey data or pregnancy weight measured after randomization was available. "

12b) Methods for additional analyses, such as subgroup analyses and adjusted analyses

Does your paper address CONSORT subitem 12b? *

Copy and paste relevant sections from the manuscript (include quotes in quotation marks "like this" to indicate direct quotes from your manuscript), or elaborate on this item by providing additional information not in the ms, or briefly explain why the item is not applicable/relevant for your study

"All analyses were adjusted for the variables used in the adaptive randomization procedure as well as pre-pregnancy weight for gestational weight gain outcomes and baseline physical activity for physical activity outcomes. Therefore, our adjusted model for change in physical activity adjusted for physical activity at baseline survey, age, parity, pre-pregnancy BMI, race/ethnicity, and difference in GA weeks between baseline survey and the second survey. The adjusted model for GWG adjusted for age, parity, pre-pregnancy BMI, race/ethnicity, and difference in GA weeks between last and first measured weight during pregnancy."

X26) REB/IRB Approval and Ethical Considerations [recommended as subheading under "Methods"] (not a CONSORT item)

X26-i) Comment on ethics committee approval

	1	2	3	4	5	
subitem not at all important	○	○	○	◉	○	essential

Clear selection

Does your paper address subitem X26-i?

Copy and paste relevant sections from the manuscript (include quotes in quotation marks "like this" to indicate direct quotes from your manuscript), or elaborate on this item by providing additional information not in the ms, or briefly explain why the item is not applicable/relevant for your study

"STRIDE was approved by the Kaiser Permanente Northern California Institutional Review Board."

x26-ii) Outline informed consent procedures

Outline informed consent procedures e.g., if consent was obtained offline or online (how? Checkbox, etc.?), and what information was provided (see 4a-ii). See [6] for some items to be included in informed consent documents.

	1	2	3	4	5	
subitem not at all important	○	◉	○	○	○	essential

Clear selection

Does your paper address subitem X26-ii?

Copy and paste relevant sections from the manuscript (include quotes in quotation marks "like this" to indicate direct quotes from your manuscript), or elaborate on this item by providing additional information not in the ms, or briefly explain why the item is not applicable/relevant for your study

Your answer

X26-iii) Safety and security procedures

Safety and security procedures, incl. privacy considerations, and any steps taken to reduce the likelihood or detection of harm (e.g., education and training, availability of a hotline)

	1	2	3	4	5	
subitem not at all important	○	○	◉	○	○	essential

Clear selection

Does your paper address subitem X26-iii?

Copy and paste relevant sections from the manuscript (include quotes in quotation marks "like this" to indicate direct quotes from your manuscript), or elaborate on this item by providing additional information not in the ms, or briefly explain why the item is not applicable/relevant for your study

Your answer

RESULTS

13a) For each group, the numbers of participants who were randomly assigned, received intended treatment, and were analysed for the primary outcome

NPT: The number of care providers or centers performing the intervention in each group and the number of patients treated by each care provider in each center

Does your paper address CONSORT subitem 13a? *

Copy and paste relevant sections from the manuscript (include quotes in quotation marks "like this" to indicate direct quotes from your manuscript), or elaborate on this item by providing additional information not in the ms, or briefly explain why the item is not applicable/relevant for your study

"Among 90 eligible patients, 83% consented to be randomized (n=75). Following randomization, 89% of intervention participants (n=35) and 92% of usual care participants completed all study surveys (n=35); and 89% of intervention participants (n=32) and 84% of usual care participants (n=32) had a weight measurement at the end

13b) For each group, losses and exclusions after randomisation, together with reasons

Does your paper address CONSORT subitem 13b? (NOTE: Preferably, this is shown in a CONSORT flow diagram) *

Copy and paste relevant sections from the manuscript (include quotes in quotation marks "like this" to indicate direct quotes from your manuscript), or elaborate on this item by providing additional information not in the ms, or briefly explain why the item is not applicable/relevant for your study

"Reasons for loss to follow-up included pregnancy loss, change to insurance, and maximum contact attempts exceeded."

13b-i) Attrition diagram

Strongly recommended: An attrition diagram (e.g., proportion of participants still logging in or using the intervention/comparator in each group plotted over time, similar to a survival curve) or other figures or tables demonstrating usage/dose/engagement.

	1	2	3	4	5	
subitem not at all important	○	○	○	◉	○	essential

Clear selection

Does your paper address subitem 13b-i?

Copy and paste relevant sections from the manuscript or cite the figure number if applicable (include quotes in quotation marks "like this" to indicate direct quotes from your manuscript), or elaborate on this item by providing additional information not in the ms, or briefly explain why the item is not applicable/relevant for your study

Yes, this information is in Figure 1.

14a) Dates defining the periods of recruitment and follow-up

Does your paper address CONSORT subitem 14a? *

Copy and paste relevant sections from the manuscript (include quotes in quotation marks "like this" to indicate direct quotes from your manuscript), or elaborate on this item by providing additional information not in the ms, or briefly explain why the item is not applicable/relevant for your study

"conducted between May 2017 and May 2018"

14a-i) Indicate if critical “secular events” fell into the study period

Indicate if critical “secular events” fell into the study period, e.g., significant changes in Internet resources available or “changes in computer hardware or Internet delivery resources”

	1	2	3	4	5	
subitem not at all important	○	○	◉	○	○	essential

Clear selection

Does your paper address subitem 14a-i?

Copy and paste relevant sections from the manuscript (include quotes in quotation marks "like this" to indicate direct quotes from your manuscript), or elaborate on this item by providing additional information not in the ms, or briefly explain why the item is not applicable/relevant for your study

No critical “secular events” fell into the study period

14b) Why the trial ended or was stopped (early)

Does your paper address CONSORT subitem 14b? *

Copy and paste relevant sections from the manuscript (include quotes in quotation marks "like this" to indicate direct quotes from your manuscript), or elaborate on this item by providing additional information not in the ms, or briefly explain why the item is not applicable/relevant for your study

Our trial was not stopped early

15) A table showing baseline demographic and clinical characteristics for each group

NPT: When applicable, a description of care providers (case volume, qualification, expertise, etc.) and centers (volume) in each group

Does your paper address CONSORT subitem 15? *

Copy and paste relevant sections from the manuscript (include quotes in quotation marks "like this" to indicate direct quotes from your manuscript), or elaborate on this item by providing additional information not in the ms, or briefly explain why the item is not applicable/relevant for your study

Yes, this information was included in Table 2

15-i) Report demographics associated with digital divide issues

In ehealth trials it is particularly important to report demographics associated with digital divide issues, such as age, education, gender, social-economic status, computer/Internet/ehealth literacy of the participants, if known.

	1	2	3	4	5	
subitem not at all important	○	○	◉	○	○	essential

Clear selection

Does your paper address subitem 15-i? *

Copy and paste relevant sections from the manuscript (include quotes in quotation marks "like this" to indicate direct quotes from your manuscript), or elaborate on this item by providing additional information not in the ms, or briefly explain why the item is not applicable/relevant for your study

Yes, this information was included in Table 2

16) For each group, number of participants (denominator) included in each analysis and whether the analysis was by original assigned groups

16-i) Report multiple “denominators” and provide definitions

Report multiple “denominators” and provide definitions: Report N’s (and effect sizes) “across a range of study participation [and use] thresholds” [1], e.g., N exposed, N consented, N used more than x times, N used more than y weeks, N participants “used” the intervention/comparator at specific pre-defined time points of interest (in absolute and relative numbers per group). Always clearly define “use” of the intervention.

	1	2	3	4	5	
subitem not at all important	○	○	◉	○	○	essential

Clear selection

Does your paper address subitem 16-i? *

Copy and paste relevant sections from the manuscript (include quotes in quotation marks "like this" to indicate direct quotes from your manuscript), or elaborate on this item by providing additional information not in the ms, or briefly explain why the item is not applicable/relevant for your study

"Among 90 eligible patients, 83% consented to be randomized (n=75). Following randomization, 89% of intervention participants (n=35) and 92% of usual care participants completed all study surveys (n=35); and 89% of intervention participants (n=32) and 84% of usual care participants (n=32) had a weight measurement at the end

16-ii) Primary analysis should be intent-to-treat

Primary analysis should be intent-to-treat, secondary analyses could include comparing only “users”, with the appropriate caveats that this is no longer a randomized sample (see 18-i).

	1	2	3	4	5	
subitem not at all important	○	○	◉	○	○	essential

Clear selection

Does your paper address subitem 16-ii?

Copy and paste relevant sections from the manuscript (include quotes in quotation marks "like this" to indicate direct quotes from your manuscript), or elaborate on this item by providing additional information not in the ms, or briefly explain why the item is not applicable/relevant for your study

Yes, we used an intent-to-treat analysis

17a) For each primary and secondary outcome, results for each group, and the estimated effect size and its precision (such as 95% confidence interval)

Does your paper address CONSORT subitem 17a? *

Copy and paste relevant sections from the manuscript (include quotes in quotation marks "like this" to indicate direct quotes from your manuscript), or elaborate on this item by providing additional information not in the ms, or briefly explain why the item is not applicable/relevant for your study

"While this pilot study was not powered for clinical outcomes, in exploratory analyses, we found that participants in the intervention had greater change in total activity per week compared to usual care (Table 4). Participants in the intervention arm had a 23.46 MET-hours greater change in self-reported total physical activity per week (95% CI:1.13, 45.8) and a 247.2 minute greater change in moderate intensity physical activity per week (95% CI: 36.2, 530.6) in unadjusted models, but this effect was attenuated in adjusted models (change in total physical activity: 15.55 MET-hours per week; 95% CI: -6.32, 37.42; change in moderate intensity physical activity: 199.6 minutes per week; 95% CI: -43.7, 442.9). We found no difference between arms in total gestational weight gain (mean difference: 1.14 kg; 95% CI: -0.71, 3.00) or rate of gestational weight gain (mean difference; 0.03 kg; 95% CI: -0.02, 0.09). "

17a-i) Presentation of process outcomes such as metrics of use and intensity of use

In addition to primary/secondary (clinical) outcomes, the presentation of process outcomes such as metrics of use and intensity of use (dose, exposure) and their operational definitions is critical. This does not only refer to metrics of attrition (13-b) (often a binary variable), but also to more continuous exposure metrics such as "average session length". These must be accompanied by a technical description how a metric like a "session" is defined (e.g., timeout after idle time) [1] (report under item 6a).

	1	2	3	4	5	
subitem not at all important	○	○	○	◉	○	essential

Clear selection

Does your paper address subitem 17a-i?

Copy and paste relevant sections from the manuscript (include quotes in quotation marks "like this" to indicate direct quotes from your manuscript), or elaborate on this item by providing additional information not in the ms, or briefly explain why the item is not applicable/relevant for your study

"On average, participants in the intervention condition had a high adherence to self-monitoring: participants wore the tracker 6 days per week and weighed themselves 5 times per week."

17b) For binary outcomes, presentation of both absolute and relative effect sizes is recommended

Does your paper address CONSORT subitem 17b? *

Copy and paste relevant sections from the manuscript (include quotes in quotation marks "like this" to indicate direct quotes from your manuscript), or elaborate on this item by providing additional information not in the ms, or briefly explain why the item is not applicable/relevant for your study

Our study outcomes were not binary.

18) Results of any other analyses performed, including subgroup analyses and adjusted analyses, distinguishing pre-specified from exploratory

Does your paper address CONSORT subitem 18? *

Copy and paste relevant sections from the manuscript (include quotes in quotation marks "like this" to indicate direct quotes from your manuscript), or elaborate on this item by providing additional information not in the ms, or briefly explain why the item is not applicable/relevant for your study

". All analyses were adjusted for the variables used in the adaptive randomization procedure as well as pre-pregnancy weight for gestational weight gain outcomes and baseline physical activity for physical activity outcomes. Therefore, our adjusted model for change in physical activity adjusted for physical activity at baseline survey, age, parity, pre-pregnancy BMI, race/ethnicity, and difference in GA weeks between baseline survey and the second survey. The adjusted model for GWG adjusted for age, parity, pre-pregnancy BMI, race/ethnicity, and difference in GA weeks between last and first measured weight during pregnancy. "
"Exploratory Analyses
While this pilot study was not powered for clinical outcomes, in exploratory analyses, we found that participants in the intervention had greater change in total activity per week compared to usual care (Table 4). Participants in the intervention arm had a 23.46 MET-hours greater change in self-reported total physical activity per week (95% CI:1.13, 45.8) and a 247.2 minute greater change in moderate intensity physical activity per week (95% CI: 36.2, 530.6) in unadjusted models, but this effect was attenuated in adjusted models (change in total physical activity: 15.55 MET-hours per week; 95% CI: -6.32, 37.42; change in moderate intensity physical activity: 199.6 minutes per week; 95% CI: -43.7, 442.9). We found no difference between arms in total gestational weight gain (mean difference: 1.14 kg; 95% CI: -0.71, 3.00) or rate of gestational weight gain (mean difference; 0.03 kg; 95% CI: -0.02, 0.09). In addition, we did not find, and were not powered to find, any significant differences between intervention and usual care study arms in any adverse perinatal outcomes (data not shown). "

18-i) Subgroup analysis of comparing only users

A subgroup analysis of comparing only users is not uncommon in ehealth trials, but if done, it must be stressed that this is a self-selected sample and no longer an unbiased sample from a randomized trial (see 16-iii).

	1	2	3	4	5	
subitem not at all important	○	◉	○	○	○	essential

Clear selection

Does your paper address subitem 18-i?

Copy and paste relevant sections from the manuscript (include quotes in quotation marks "like this" to indicate direct quotes from your manuscript), or elaborate on this item by providing additional information not in the ms, or briefly explain why the item is not applicable/relevant for your study

Your answer

19) All important harms or unintended effects in each group

(for specific guidance see CONSORT for harms)

Does your paper address CONSORT subitem 19? *

Copy and paste relevant sections from the manuscript (include quotes in quotation marks "like this" to indicate direct quotes from your manuscript), or elaborate on this item by providing additional information not in the ms, or briefly explain why the item is not applicable/relevant for your study

There were no important harms or unintended effects.

19-i) Include privacy breaches, technical problems

Include privacy breaches, technical problems. This does not only include physical “harm” to participants, but also incidents such as perceived or real privacy breaches [1], technical problems, and other unexpected/unintended incidents. “Unintended effects” also includes unintended positive effects [2].

	1	2	3	4	5	
subitem not at all important	○	○	◉	○	○	essential

Clear selection

Does your paper address subitem 19-i?

Copy and paste relevant sections from the manuscript (include quotes in quotation marks "like this" to indicate direct quotes from your manuscript), or elaborate on this item by providing additional information not in the ms, or briefly explain why the item is not applicable/relevant for your study

Your answer

19-ii) Include qualitative feedback from participants or observations from staff/researchers

Include qualitative feedback from participants or observations from staff/researchers, if available, on strengths and shortcomings of the application, especially if they point to unintended/unexpected effects or uses. This includes (if available) reasons for why people did or did not use the application as intended by the developers.

	1	2	3	4	5	
subitem not at all important	○	○	○	◉	○	essential

Clear selection

Does your paper address subitem 19-ii?

Copy and paste relevant sections from the manuscript (include quotes in quotation marks "like this" to indicate direct quotes from your manuscript), or elaborate on this item by providing additional information not in the ms, or briefly explain why the item is not applicable/relevant for your study

"Twenty-two participants agreed to be contacted for focus groups and were invited to participate in the qualitative study via email recruitment. A total of fourteen participants enrolled in three focus groups (64% response rate). Focus groups lasted 40-50 minutes. Table 3 lists the ratings of each intervention component and illustrative quotes related to the component. The digital scale was rated most highly (97% of participants rated very or moderately helpful) among all the intervention components followed by the Coach calls (79% of participants rated very or moderately helpful), physical activity tracker and text messages (73% of participants rated very or moderately helpful), with the mHealth website rated lowest (58% of participants rated very or moderately helpful). Overall, the intervention was well received, and focus group participants reported that the mHealth lifestyle intervention helped them in two main ways. First, the intervention promoted accountability. Secondly, the intervention motivated them and provided tangible support toward physical activity goals. Similarly, the most highly rated intervention components (i.e., digital scale, Coach calls, physical activity tracker and text messages) were cited as the most useful mechanisms for both accountability and motivation. The mHealth website was viewed more as a place to see all activity tracked in one place, but less of a mechanism toward promoting accountability or motivation toward goals. Participants reported enjoying the mHealth lifestyle intervention program and believed that the program led to more physical activity and less gestational weight

DISCUSSION

22) Interpretation consistent with results, balancing benefits and harms, and considering other relevant evidence

NPT: In addition, take into account the choice of the comparator, lack of or partial blinding, and unequal expertise of care providers or centers in each group

22-i) Restate study questions and summarize the answers suggested by the data, starting with primary outcomes and process outcomes (use)

Restate study questions and summarize the answers suggested by the data, starting with primary outcomes and process outcomes (use).

	1	2	3	4	5	
subitem not at all important	○	○	○	◉	○	essential

Clear selection

Does your paper address subitem 22-i? *

Copy and paste relevant sections from the manuscript (include quotes in quotation marks "like this" to indicate direct quotes from your manuscript), or elaborate on this item by providing additional information not in the ms, or briefly explain why the item is not applicable/relevant for your study

"In this mixed-methods acceptability and feasibility randomized controlled pilot trial, we found that a mHealth intervention for pregnant patients with overweight or obesity was feasible and acceptable for participants and successfully promoted weight and physical activity self-monitoring. There was a high level of adherence to the self-monitoring of weight and physical activity among participants in the intervention arm and overall participants rated the program highly. Focus groups found participants desired more support related to nutrition and a more mobile-friendly app instead of a mHealth website. In exploratory analyses, we found that the mHealth lifestyle intervention increased minutes of physical activity per week compared to usual care but observed no difference in gestational weight gain."

22-ii) Highlight unanswered new questions, suggest future research

Highlight unanswered new questions, suggest future research.

	1	2	3	4	5	
subitem not at all important	○	○	○	◉	○	essential

Clear selection

Does your paper address subitem 22-ii?

Copy and paste relevant sections from the manuscript (include quotes in quotation marks "like this" to indicate direct quotes from your manuscript), or elaborate on this item by providing additional information not in the ms, or briefly explain why the item is not applicable/relevant for your study

"Our study demonstrates that the use of mHealth technology to deliver a theory-based lifestyle intervention is acceptable for pregnant patients with overweight or obesity. One goal of this pilot trial was to inform the design of a larger randomized controlled trial. To this end, the study team is currently implementing a pragmatic cluster randomized clinical trial that expands the pilot intervention based on participant feedback to incorporate additional nutrition support via nutrition self-monitoring and implements an app-based version of the pilot mHealth website to support better utilization. More effective interventions with broad reach are needed to help pregnant patients with overweight or obesity increase physical activity and meet the IOM guidelines for gestational weight gain."

20) Trial limitations, addressing sources of potential bias, imprecision, and, if relevant, multiplicity of analyses

20-i) Typical limitations in ehealth trials

Typical limitations in ehealth trials: Participants in ehealth trials are rarely blinded. Ehealth trials often look at a multiplicity of outcomes, increasing risk for a Type I error. Discuss biases due to non-use of the intervention/usability issues, biases through informed consent procedures, unexpected events.

	1	2	3	4	5	
subitem not at all important	○	○	○	●	○	essential

Clear selection

Does your paper address subitem 20-i? *

Copy and paste relevant sections from the manuscript (include quotes in quotation marks "like this" to indicate direct quotes from your manuscript), or elaborate on this item by providing additional information not in the ms, or briefly explain why the item is not applicable/relevant for your study

"As with any study, ours has several limitations. First, this was a small randomized controlled pilot trial to assess the intervention's acceptability and feasibility. As such, our study was not powered to detect clinical outcomes. Second, we relied on self-reported measures of physical activity, although the PPAQ is a validated self-report tool for physical activity during pregnancy. Third, pregnant patients randomized to the usual care arm had higher levels of baseline physical activity than women randomized to the intervention arm; however, we adjusted for baseline differences in our analysis. Fourth, this study utilized BMI measurements which on its own, has limitations. However, this was the data available in the electronic health record and BMI is still widely used clinically due to its ease of measurement. Finally, participants were not masked to study group, which could have led to biased reports in the intervention arm."

21) Generalisability (external validity, applicability) of the trial findings

NPT: External validity of the trial findings according to the intervention, comparators, patients, and care providers or centers involved in the trial

21-i) Generalizability to other populations

Generalizability to other populations: In particular, discuss generalizability to a general Internet population, outside of a RCT setting, and general patient population, including applicability of the study results for other organizations

	1	2	3	4	5	
subitem not at all important	○	○	○	○	○	essential

Does your paper address subitem 21-i?

Copy and paste relevant sections from the manuscript (include quotes in quotation marks "like this" to indicate direct quotes from your manuscript), or elaborate on this item by providing additional information not in the ms, or briefly explain why the item is not applicable/relevant for your study

Your answer

21-ii) Discuss if there were elements in the RCT that would be different in a routine application setting

Discuss if there were elements in the RCT that would be different in a routine application setting (e.g., prompts/reminders, more human involvement, training sessions or other co-interventions) and what impact the omission of these elements could have on use, adoption, or outcomes if the intervention is applied outside of a RCT setting.

	1	2	3	4	5	
subitem not at all important	○	○	○	○	○	essential

Does your paper address subitem 21-ii?

Copy and paste relevant sections from the manuscript (include quotes in quotation marks "like this" to indicate direct quotes from your manuscript), or elaborate on this item by providing additional information not in the ms, or briefly explain why the item is not applicable/relevant for your study

Your answer

OTHER INFORMATION

23) Registration number and name of trial registry

Does your paper address CONSORT subitem 23? *

Copy and paste relevant sections from the manuscript (include quotes in quotation marks "like this" to indicate direct quotes from your manuscript), or elaborate on this item by providing additional information not in the ms, or briefly explain why the item is not applicable/relevant for your study

"Trial Registration: ClinicalTrials.gov NCT03936283"

24) Where the full trial protocol can be accessed, if available

Does your paper address CONSORT subitem 24? *

Cite a Multimedia Appendix, other reference, or copy and paste relevant sections from the manuscript (include quotes in quotation marks "like this" to indicate direct quotes from your manuscript), or elaborate on this item by providing additional information not in the ms, or briefly explain why the item is not applicable/relevant for your study

this is a feasibility pilot study so we have not included a full trial protocol.

25) Sources of funding and other support (such as supply of drugs), role of funders

Does your paper address CONSORT subitem 25? *

Copy and paste relevant sections from the manuscript (include quotes in quotation marks "like this" to indicate direct quotes from your manuscript), or elaborate on this item by providing additional information not in the ms, or briefly explain why the item is not applicable/relevant for your study

"Acknowledgments: This study was funded by the Delivery Science Grants Program, The Permanente Medical Group. Dr. Thomas received funding from The Permanente Medical Group Delivery Science Fellowship Program and the National Institute of Diabetes and Digestive and Kidney Diseases grant T32DK11668401. Dr. Badon received funding from the Eunice Kennedy Shriver National Institute of Child Health and Human Development grant K99HD100585 and Kaiser Permanente Northern California Community Health. Dr. Hedderson received funding from R01DK118455 to conduct this study. Dr. Brown received funding from the National Institute of Diabetes and Digestive and Kidney Diseases grant K01 DK099404. "

X27) Conflicts of Interest (not a CONSORT item)

X27-i) State the relation of the study team towards the system being evaluated

In addition to the usual declaration of interests (financial or otherwise), also state the relation of the study team towards the system being evaluated, i.e., state if the authors/evaluators are distinct from or identical with the developers/sponsors of the intervention.

	1	2	3	4	5	
subitem not at all important	○	○	◉	○	○	essential

Clear selection

Does your paper address subitem X27-i?

Copy and paste relevant sections from the manuscript (include quotes in quotation marks "like this" to indicate direct quotes from your manuscript), or elaborate on this item by providing additional information not in the ms, or briefly explain why the item is not applicable/relevant for your study

Your answer

About the CONSORT EHEALTH checklist

As a result of using this checklist, did you make changes in your manuscript? *

- ◉ yes, major changes
- ○ yes, minor changes
- ○ no

What were the most important changes you made as a result of using this checklist?

Your answer

How much time did you spend on going through the checklist INCLUDING making changes in your manuscript *

five (5) hours to complete

As a result of using this checklist, do you think your manuscript has improved? *

- (selected) yes
- no
- Other:

Would you like to become involved in the CONSORT EHEALTH group?

This would involve for example becoming involved in participating in a workshop and writing an "Explanation and Elaboration" document

- yes
- (selected) no
- Other:

Clear selection

Any other comments or questions on CONSORT EHEALTH

Your answer

STOP - Save this form as PDF before you click submit

To generate a record that you filled in this form, we recommend to generate a PDF of this page (on a Mac, simply select "print" and then select "print as PDF") before you submit it.

When you submit your (revised) paper to JMIR, please upload the PDF as supplementary file.

Don't worry if some text in the textboxes is cut off, as we still have the complete information in our database. Thank you!

Final step: Click submit !

Click submit so we have your answers in our database!

Submit

Clear form

Never submit passwords through Google Forms.

This form was created outside of your domain. Report Abuse - Terms of Service - Privacy Policy

Google Forms
